# Patterns of psychotropic medication use among young people living in out-of-home care: A scoping review and meta-analysis of international literature

**DOI:** 10.1177/00048674251370467

**Published:** 2025-09-12

**Authors:** Kostas Hatzikiriakidis, Emma Galvin, Luke Patitsas, Helen Skouteris

**Affiliations:** 1 Health and Social Care Unit, School of Public Health and Preventive Medicine, Monash University, VIC, Australia; 2 Warwick Business School, Warwick University, Coventry, UK

**Keywords:** Young people, out-of-home care, mental health, psychotropic medication

## Abstract

**Background:**

Young people in out-of-home care experience complex mental health needs and may be prescribed psychotropic medications at a greater rate than those not living in care. The aim of this scoping review was to synthesise international literature to (1) understand the prevalence of psychotropic medication use among young people in out-of-home care and (2) identify the factors associated with a greater likelihood of prescribing and/or use.

**Methods:**

This scoping review was conducted according to the Joanna Briggs Institute (JBI) methodological guidance. Five electronic databases were searched for relevant literature published from inception to September 2024. Synthesising the literature involved a mixed-method approach, utilising a proportional meta-analysis, narrative synthesis and content analysis.

**Results:**

Sixty-one studies were eligible for inclusion. Meta-analysis calculated the pooled prevalence of any psychotropic medication as 42.16% (95% confidence interval [CI]: 31.76–52.93%). Pooled prevalence estimates for individual subclasses were 25.60% for stimulants (16.82–35.51%), 21.33% for antipsychotics (12.42–31.87%), 16.36% for antidepressants (10.35–23.42%), 8.57% for mood stabilisers (4.61–13.58%) and 2.24% for anxiolytics (1.12–3.72%). The most commonly examined predisposing factors suggested differences in prescribing practices associated with demographic characteristics such as age, sex and ethnicity.

**Conclusions:**

Psychotropic medication management in out-of-home care is complex; however, further research on the international prescribing practices outside the United States is needed. Improved cross-system coordination, caregiver support, meaningful youth involvement and trauma-informed, person-centred approaches to mental health care in out-of-home care are essential to ensure safe, effective and equitable psychotropic medication use.

## Introduction

Out-of-home care^1^ is a term that encompasses various forms of substitute care for young people who have been removed from their families of origin in response to the presence of ongoing maltreatment, abuse and/or neglect (Australian Institute of Health and Welfare, 2024). The United Nations’ *Guidelines for the Alternative Care of Children* differentiates between several types of care arrangements for young people, including foster care, kinship care, residential care and supervised independent living (United Nations, 2010). Placement in out-of-home care is designed to support a young person until they transition into independent living at the age of 18 years (or 21 years in some jurisdictions) (Mendes and Rogers, 2020; Stein, 2019).

Given the complex histories of trauma observed among this population, young people living in out-of-home care experience greater mental health needs than those not living in care (Dubois-Comtois et al., 2021). In particular, research has documented an increased prevalence of diagnosed mental health conditions (Bronsard et al., 2016; Engler et al., 2020; Tarren-Sweeney, 2008), alongside a greater risk of experiencing psychological distress, post-traumatic stress, substance use, self-harm and suicidal ideation (Evans et al., 2017; Maclean et al., 2016). These adverse life outcomes can have a profound and lasting impact on their overall development and lead to other adverse experiences after transitioning from care (e.g. educational disengagement unemployment, homelessness, social isolation, criminal justice involvement) (Baidawi et al., 2014).

Although many young people in out-of-home care exhibit a heightened need for access to mental health services and therapeutic interventions, evidence indicates that these support needs often remain unmet (Pecora et al., 2009; Petrenko et al., 2011). This gap in service provision may result in a reliance on the use of psychotropic medications to treat emotional and behavioural needs (Longhofer et al., 2011).

Psychotropic medications refer to drugs that affect an individual’s thoughts, emotions and behaviours and are often prescribed to manage the symptoms of various mental health conditions (Ernstmeyer and Christman, 2022). Research exploring the use of psychotropic medications has broadly classified them into several major classes, including antidepressants, antipsychotics, anxiolytics, mood stabilisers and stimulants, all of which have distinct therapeutic purposes and mechanisms of action. For example, stimulants such as amphetamines and methylphenidate are often prescribed to children to manage symptoms of attention-deficit hyperactivity disorder (ADHD), such as reducing hyperactivity, regulating attention and controlling impulsivity (Cortese et al., 2018).

In contrast, first- and second-generation antipsychotics are commonly prescribed for the treatment of symptoms associated with psychosis (e.g. delusions, hallucinations) in conditions such as schizophrenia (Krause et al., 2018). Although psychotropic medications are often prescribed as part of a broader mental health treatment plan that is tailored to an individual’s needs, the decision to prescribe psychotropic medications is complex and requires careful consideration of the benefits against the risks, especially in children and adolescents. In particular, evidence suggests that excessive use of antipsychotics in children and adolescents presents an elevated risk of adverse physiological and cardiometabolic abnormalities that can have a long-term impact on their developmental health and wellbeing (De Hert et al., 2011; Pisano et al., 2016; Rogdaki et al., 2024).

While psychotropic medications can form an integral role in managing a young person’s symptoms, concerns have been raised regarding their overutilisation in out-of-home care and whether they are being prescribed to young people in a safe and appropriate manner (Longhofer et al., 2011). In the United States, federal inquiries have revealed that young people in care are prescribed psychotropic medications at disproportionate rates when compared to young people who are not care experienced (Government Accountability Office, 2011). These inquiries have also drawn particular attention towards increases in antipsychotic prescriptions, greater rates of polypharmacy (i.e. concomitant prescription of two or more psychotropic medications) and prescribed doses that are higher than recommended levels, raising concerns about the complexity of treatment and the potential for adverse long-term side effects.

In addition, foster caregivers, caseworkers, clinicians and young people with a lived experience of care in the United States have previously expressed that off-label psychotropic medications are often overprescribed to manage or control maladaptive behaviours, rather than first exploring and addressing the presence of underlying trauma (Barnett et al., 2016a; Bowden et al., 2022). However, international evidence from other countries is limited and highlights a need for more comprehensive data on global prescribing patterns for this population.

In response to the overutilisation of psychotropic medication among young people in care, research has recognised the importance of systems that promote adherence to appropriate prescribing and medication-management practices (Government Accountability Office, 2017). For example, Mackie et al. (2022) developed a framework that outlined the procedural elements of informed consent for the prescribing of psychotropic medications to youth in foster care and conducted a legislative review of child welfare policies across 50 U.S. states. The framework included five core elements: (1) gather the child’s social and medical history; (2) specification of the activities required during clinical encounters to prescribe medication (e.g. mental health assessment, providing information about the medication, laboratory tests, side effect monitoring); (3) authorise the psychotropic medication by a designated decision-maker and/or youth assessment; (4) notify relevant stakeholders of the decision to prescribe (e.g. caseworker, parents, guardians, etc.) and (5) ongoing review of the decision to prescribe. However, only two states maintained policies that captured all five elements of the framework, showing clear differences in how policies and procedures are applied across states. This inconsistency raises concerns about whether proper safeguards are implemented to ensure that psychotropic medications are prescribed appropriately for young people in care (Mackie et al., 2022).

Given these interstate variations in the legislative endorsement of consistent prescribing practices and the risks associated with an overreliance on psychotropic medication in young people, research has recognised the importance of identifying the factors that might contribute to an elevated likelihood of prescribing psychotropic medications to young people in out-of-home care. In a recent scoping review of predisposing factors associated with psychotropic medication use in out-of-home care across the United States, Xu et al. (2023) found that higher prescribing rates have been observed among young people who were older, male, White, had a history of abuse and lived in congregate care settings, suggesting that prescribing practices are not only shaped by clinical need. Despite the need for effective medication management for young people in out-of-home care, existing research has largely been conducted in the Unites States, and gaps remain in our understanding of the international prevalence of medication use and the factors associated with prescribing practices. Therefore, the aim of this scoping review was to synthesise international literature to (1) understand the prevalence of psychotropic medication use among young people in out-of-home care and (2) identify the factors associated with a greater likelihood of prescribing and/or use.

## Method

### Study design

A scoping review was guided by the Joanna Briggs Institute (JBI) methodological guidance for conducting scoping reviews (Peters et al., 2020) and reported in accordance with the PRISMA Extension for Scoping Reviews Checklist (PRISMA-ScR) (Tricco et al., 2018). The protocol was registered prospectively in Open Science Framework (https://doi.org/10.17605/OSF.IO/YQ495).

### Search strategy

A search for relevant literature was conducted in September 2024. The following academic databases were searched: MEDLINE, Scopus, EMBASE, PsycINFO and CINAHL Plus. The search strategy was guided by the Population, Context, Concept (PCC) framework (Peters et al., 2020) and developed using the following combination of keywords, relevant synonyms and MeSH terms: ‘young people’ (Population), ‘out-of-home care’ (Context) and ‘medication’ (Concept) (see Supplemental Material).

### Study selection

The search results were exported and managed using the review management software, Covidence (Veritas Health Innovation, 2023). A summary of the inclusion and exclusion criteria has been provided in Table 1. Following the exclusion of duplicates, the titles and abstracts of all articles were independently screened by two reviewers (K.H. and L.P.). Articles that were not excluded at this stage were then read in full and screened against the inclusion and exclusion criteria by the same two reviewers. Any discrepancies were resolved via discussion, and a third reviewer (E.G.) was available if consensus could not be reached. The interrater reliability, calculated as the proportion of studies that received the same initial rating by both reviewers at the title/abstract and full-text stages, was 97.78% and 89.84%, respectively.

**Table 1.** Summary of inclusion and exclusion criteria.

	Inclusion criteria	Exclusion criteria
Population (P)	Children, adolescents and young people up to the age of 18–21 years.	Adults over the age of 21 years who do not have a lived experience of out-of-home care.
Context (C)	Any form of out-of-home care, including residential, foster and kinship care arrangements (given international differences in terminology, out-of-home care was defined as any form of statutory care for young people who are unable to live with their own families due to the presence of abuse, maltreatment or neglect).	Any context or setting other than out-of-home care, including young people living with their own families in the community, or other residential settings such as temporary residential mental health treatment programmes.
Concept (C)	Studies that explore either (1) the prevalence of medication prescribing and use (including polypharmacy); and (2) predisposing factors associated with medication use.	Studies that do not focus on medication management for young people in out-of-home care.
Study design and article type	Any quantitative study design (e.g., cross-sectional, cohort studies, randomised controlled trials, etc.) or mixed-method studies.	Qualitative study designs or articles that did not report the results of a primary research study (e.g., literature reviews, study protocols, commentaries, editorials, perspective pieces and conference abstracts without an associated full text).
Language	Articles written in English language.	Articles written in languages other than English.
Country	Research conducted in any country was considered eligible for inclusion.	No limitation on country was applied.
Timeframe	Articles published since database inception, up until September 2024.	Articles published after the updated search was conducted in September 2024.

### Data extraction and analysis

Data extraction of all included studies was performed by one researcher (K.H.), with 10% of studies independently co-extracted by a second researcher (L.P.). Descriptive data extracted from articles included: first author; publication year; country; study design; placement type (i.e. residential, foster or kinship care); participant group and demographics (age, sex, race/ethnicity and mental health diagnoses). Methodological quality and risk of bias were not assessed, as this scoping review aimed to map the available evidence, regardless of methodological quality (Peters et al., 2020).

For the first aim, prevalence data on psychotropic medication use in young people in out-of-home care and comparison groups were extracted, including data for individual medication classes and polypharmacy rates. To synthesise the prevalence of psychotropic medication use, a proportional meta-analysis was conducted (Barker et al., 2021; Wang, 2023). Studies were eligible for meta-analysis if they reported the overall sample size and included clear data on the number of young people in care who were prescribed medication. Prevalence data were normalised using a double arcsine transformation (Lin and Xu, 2020), and a random effects model was applied as significant heterogeneity was expected (Riley et al., 2011). The pooled prevalence estimate was reported as a mean with a 95% confidence interval (CI) and presented in a series of forest plots. Heterogeneity was assessed using the *I*^2^ statistic (Higgins et al., 2003). The influence of publication bias was not assessed as researchers have previously acknowledged that established tests for assessing publication bias have limited utility in a meta-analysis of proportions (Barker et al., 2021; Wang, 2023). Statistical analyses were conducted using *R* version 4.1.1 (R Core Team, 2021), with the *meta* (version 7.0-0) and *metafor* (version 4.6-0) packages (Balduzzi et al., 2019; Viechtbauer, 2010).

For the second aim, potential predictors of medication use were extracted alongside relevant descriptive and inferential statistics. Data were presented in summary tables and reported using narrative synthesis (Popay et al., 2006).

## Results

### Study selection

**Figure 1.**
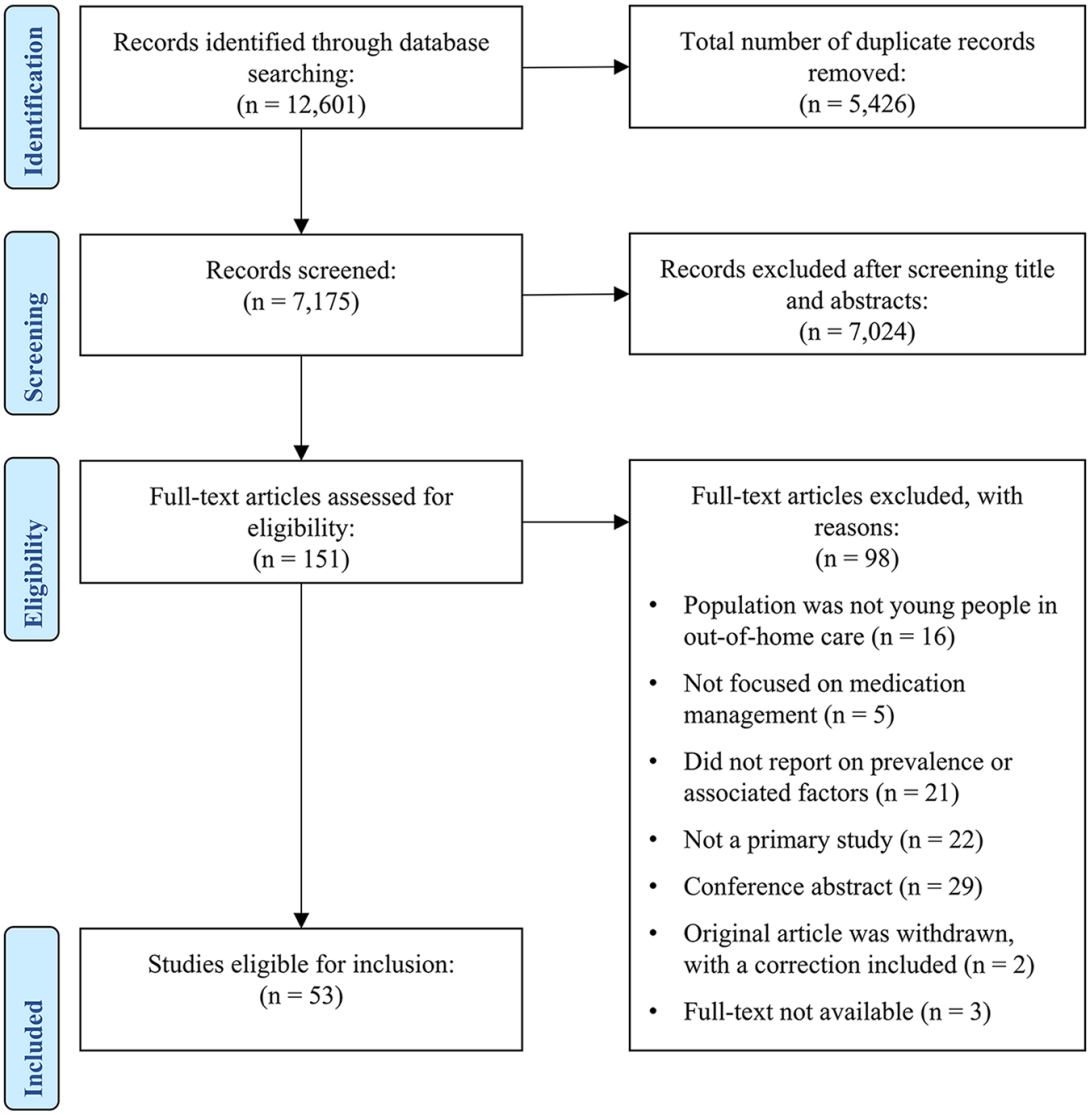
Screening flowchart illustrating the study-selection process.

The stages of study selection have been presented in Figure 1. The search identified a total of 12,601 articles, and 5426 duplicates were removed, leaving 7175 articles to assess at the title and abstract stage, where 7024 articles were excluded. The remining 151 articles were assessed at the full-text stage, where 98 articles were excluded. A total of 53 articles, representing 52 studies, were therefore included.

### Study and participant characteristics

An aggregated summary of study characteristics is provided in Table 2, and one with greater detail with a summary of the sample demographic characteristics for each study has been provided in Supplemental Table 1. Most of the included study designs were either cross-sectional (*n* = 28, 52.83%) or retrospective cohort studies (*n* = 24, 45.28%) that analysed data drawn from national and/or state-level administrative child welfare, mental health services, electronic health records and pharmaceutical insurance claims databases. Almost all studies were conducted in the United States (*n* = 49, 92.45%), with most studies representative of youth in foster care.

**Table 2.** Characteristics of included studies.

Study characteristics	Categories	Number of studies (%)
Publication year	1990–20002001–20102011–20202021–Present	2 (3.77%)11 (20.75%)23 (43.40%)17 (32.08%)
Country of publication	United StatesNorwayCanadaSpain	49 (92.45%)2 (3.77%)1 (1.89%)1 (1.89%)
Study design	Cross-sectional/descriptive studyRetrospective/prospective cohort studyRandomised controlled trial	28 (52.83%)24 (45.28%)1 (1.89%)
Setting	Foster care^a^Residential/congregate care settingsPsychiatric ward/emergency departments^b^	46 (86.79%)5 (9.43%)2 (3.77%)

a Most studies representative of foster care, where young people in state custody are looked after by nonrelative foster parents. However, some studies also used ‘foster care’ as an umbrella term that encompassed several types of alternative placements for young people (e.g. nonrelative foster care, relative/kinship care, group homes, residential care, etc.) (Hambrick et al., 2016).

b Participants in these studies were young people in foster care who were admitted to psychiatric wards and/or emergency departments.

The age distribution of young people varied widely; however, study samples were primarily representative of adolescents aged 10–18 years, with proportions ranging from 41% to 59%, while younger children aged 0–5 years were less frequently represented (0.26–2.5%). Most studies reported a balanced sex distribution of males and females; however, several studies reported a notable skew, with the proportion of males and females reported as high as 71.4% and 74.0%, respectively. Variation in the ethnicity and cultural background of young people was also observed, with most samples reported as predominantly White or European American (ranging from 20.0% to 61.7%) and Black or African American (ranging from 6.0% to 73.2%), while Hispanic representation was notably lower (ranging from 1.6% to 35.4%).

The prevalence of specific mental health diagnoses was reported by 30 studies. The most commonly reported diagnosis was ADHD (*n* = 21 studies; 7.0–89.29%), followed by anxiety disorders (*n* = 18 studies; 2.8–36.0%), depression (*n* = 17 studies; 5.0–37.0%) and conduct disorder (*n* = 13 studies; 2.3–35.5%).

### Medication prevalence

#### Prevalence of any psychotropic medications

**Figure 2.**
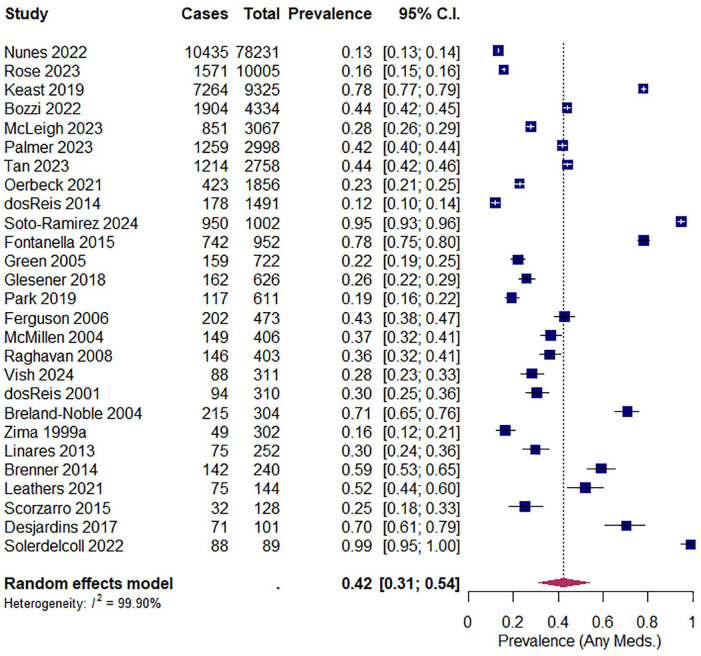
Forest plot illustrating the pooled prevalence of any psychotropic medication use across studies.

Forty-six of the 53 included studies reported the prevalence of psychotropic medication use (see Supplemental Table 2). Forty studies reported the overall prevalence of any psychotropic medication regardless of subclass, ranging from 11.9% (*n* = 178/1491) to 98.9% (*n* = 88/89). Of these studies, 27 were eligible for meta-analysis and the pooled prevalence was calculated as 42.16% (95% CI: 31.76–52.93%), with considerable heterogeneity across studies (*I*^2^ = 99.88%, 95% CI: 99.81–99.94%) (see Figure 2).

In addition, six studies compared the prevalence of any psychotropic medication between young people living in foster care and a comparison group of young people not living in care. Of these studies, three compared the prevalence of medication use between young people enrolled in different Medicaid (means-tested public health insurance) eligibility programmes and found that prevalence rates were highest among young people in foster care and those receiving Supplemental Security Income (SSI), compared to those enrolled in other programmes, such as Temporary Assistance for Needy Families (TANF) and Children’s Health Insurance Program (CHIP) (dosReis et al., 2001; Rubin et al., 2009; Zito et al., 2005).

Similarly, Keast et al. (2019) and Tan et al. (2023) found that the prevalence was highest among young people in foster care (77.9% and 44.02%, respectively) when compared to a matched comparison group of young people not living in care (62.4% and 28.02%, respectively). The final study found that medication prevalence was lowest among young people in therapeutic foster care (40%) when compared to those in Therapeutic Group Care (50%) and a Statewide Inpatient Psychiatric Program (60%) (Robst et al., 2009).

#### Prevalence of psychotropic medication subclasses

Forty-two studies provided prevalence data for individual subclasses of psychotropic medications (see Supplemental Table 2). The most commonly reported class was antipsychotics (37 studies; 2–97.70%), followed by stimulants and/or other medications for ADHD (29 studies; 5.0–92.90%), antidepressants (28 studies; 2–63%), mood stabilisers (21 studies; 1.43–42.70%) and anxiolytics (17 studies; 0.5–37.50%). Data from eligible studies underwent meta-analysis, and pooled prevalence estimates for individual medication classes were calculated as: 25.60% for stimulants and other ADHD medications (95% CI: 16.82–35.51%), 21.33% for antipsychotics (95% CI: 12.42–31.87%), 16.36% for antidepressants (95% CI: 10.35–23.42%), 8.57% for mood stabilisers (95% CI: 4.61–13.58%) and 2.24% for anxiolytics (95% CI: 1.12–3.72%). Forest plots for each subclass have been provided in the supplemental material. Fourteen studies also reported on several other subclasses, including alpha agonists, anticonvulsants, antihypertensives and hypnotics/sedatives; however, sufficient data for meta-analysis was not available.

#### Prevalence of psychotropic polypharmacy

Thirty studies reported prevalence data on polypharmacy rates (see Supplemental Table 2). Twenty of these studies reported the proportion of young people who were using two or more (1.4–61.5%), three or more (0.6–28.7%), four or more (4.8–15.0%) and five or more psychotropic medications (0.1–12.5%). Fourteen studies also provided prevalence rates for the specific number of medications young people were prescribed. Polypharmacy rates were highest for those who were prescribed two medications (1.3–80.95%), followed by those who were prescribed three (0.5–37.5%), four (0.1–25%) and five medications (0.2–12.5%). In addition, a cross-sectional study conducted by Davis et al. in one U.S. state identified that 34% of children in foster care displayed high-level polypharmacy (i.e. concurrent use of at least four classes of psychotropic medications for at least 30 days) in 2017, compared to 23% in 2012.

### Factors associated with medication use

Thirty of the 53 studies examined predisposing factors or predictors of psychotropic medication use. Dependent variables that were measured included (1) the use of any psychotropic medications (*n* = 22 studies), (2) the use of specific psychotropic medication subclasses (*n* = 13 studies) and (3) the concurrent use of multiple psychotropic medications (polypharmacy) (*n* = 7 studies). The most common independent variables included age, sex, ethnicity, psychopathology, maltreatment history, placement setting, time in care and service use (see Supplemental Table 2).

#### Age

Age was a significant predictor of psychotropic medication use across six studies; however, findings varied. Two studies found that younger children under the age of 13 years were more likely to be using psychotropic medication (Breland-Noble et al., 2004; Brenner et al., 2014), while four studies found that the likelihood of medication use was higher among older adolescents (Ferguson et al., 2006; Glesener et al., 2018; Palmer et al., 2023; Raghavan et al., 2010). In addition, two studies reported that the use of antipsychotics, stimulants and antidepressants was greater among older adolescents than among younger children (dosReis et al., 2014; Glesener et al., 2018). However, one study found that stimulant use decreased with age, while antidepressant and antipsychotic use increased (Cosme et al., 2023). One study found that the use of multiple medications was associated with being younger in age (Breland-Noble et al., 2004), another reported that concurrent medication use increased with age (Keast et al., 2019) and three studies found no association between polypharmacy and age (Brenner et al., 2014; Davis et al., 2021; Spence et al., 2019).

#### Sex

Four studies found that males were significantly more likely than females to use psychotropic medications (Ferguson et al., 2006; Leathers et al., 2021; Palmer et al., 2023; Raghavan et al., 2010); however, five studies found no differences associated with sex (Brenner et al., 2014; Glesener et al., 2018; McMillen et al., 2004; Park et al., 2019; Zima et al., 1999a). Two studies reported that males were more likely to use antipsychotics and stimulants (Glesener et al., 2018; Linares et al., 2013). The relationship between sex and polypharmacy was explored by five studies (Brenner et al., 2014; Davis et al., 2021; Keast et al., 2019; Raghavan and McMillen, 2008; Spence et al., 2019), with only one study reporting significantly higher odds of polypharmacy among males than among females (Keast et al., 2019).

#### Race/ethnicity

Eight studies found that the odds of using any psychotropic medication were consistently higher among children who were identified as White, Caucasian or European American than among Black or African American (Breland-Noble et al., 2004; Palmer et al., 2023; Raghavan et al., 2010; Zito et al., 2005), American Indian (Ferguson et al., 2006; Glesener et al., 2018) or Latino or Hispanic young people (Zima et al., 1999a) or youth of colour (McMillen et al., 2004). Two studies also reported a greater likelihood of receiving stimulants among children who were White or European American than among African American or American Indian children (dosReis et al., 2001; Glesener et al., 2018). Another study reported that African American children had significantly greater odds of antipsychotic and stimulant use than Latino or mixed-race children (Linares et al., 2013). Furthermore, two studies found that the odds of polypharmacy were significantly higher in children who were White than among children who were Black, Hispanic, American Indian or Alaskan Native, Asian or Pacific Islander (Keast et al., 2019; Raghavan and McMillen, 2008).

#### Maltreatment history

Three studies examined the association between maltreatment history and the use of any psychotropic medication, two of which found a history of neglect, physical abuse and/or sexual abuse was associated with an increased likelihood of medication use (McLeigh et al., 2023; Palmer et al., 2023). One study also examined the relationship between maltreatment history and the use of antipsychotics and stimulants and reported an increased likelihood of antipsychotic use among children with a history of sexual abuse (no other significant associations were reported) (Linares et al., 2013). In addition, one study found that adolescents with a history of physical or sexual abuse had a higher relative risk of being prescribed multiple medications than adolescents without such a history (Raghavan and McMillen, 2008).

#### Psychopathology and behaviour

The presence of mental health diagnoses and psychopathology symptoms in the clinical range were consistently reported as significant predictors of any psychotropic medication use in four studies (Brenner et al., 2014; Desjardins et al., 2017; McMillen et al., 2004; Park et al., 2019). Studies consistently reported that the likelihood of receiving medications from each class was greater for children with an existing mental health diagnosis (e.g. disruptive behaviour disorder, mood disorders, ADHD) (dosReis et al., 2014; Linares et al., 2013; Rose et al., 2023; Tan et al., 2023); however, one study also reported that young people diagnosed with schizophrenia or other psychotic disorders were more likely to use antipsychotics for a longer period of time than children with other diagnoses (Burcu et al., 2014).

In addition, young people displaying clinical levels of internalising or externalising behaviours were more likely to use medication (Breland-Noble et al., 2004; Desjardins et al., 2017; Raghavan et al., 2010) or multiple medications (Breland-Noble et al., 2004). Five studies reported associations between psychotropic polypharmacy and increasing severity of psychopathology, particularly among those with diagnoses of specific mood of neurodevelopmental disorders (e.g. major depression, bipolar, ADHD, conduct disorder, disruptive behaviour, oppositional defiant, etc.) and diagnostic comorbidity (Brenner et al., 2014; Davis et al., 2021; Keast et al., 2019; Raghavan and McMillen, 2008; Spence et al., 2019).

#### Out-of-home care placement

Although all 15 studies were conducted within the context of foster care, only four studies reported on the association between psychotropic medication use and the specific type of placement, all of which found that living in congregate, residential or group home settings was associated with greater odds of psychotropic medication use when compared to young people living with nonrelative foster parents (McMillen et al., 2004; Palmer et al., 2023; Park et al., 2019; Zima et al., 1999a).

#### Time in care

Three studies reported that the likelihood of being dispensed any psychotropic medication was associated with a younger age at the time of their initial placement into care (McMillen et al., 2004) and a longer duration of time in care (Brenner et al., 2014; Glesener et al., 2018). However, one study reported that leaving care before the age of 19 years was associated with an increased likelihood of discontinuing medication use (McMillen et al., 2004). Another study reported that a longer duration of time in care was significantly associated with an increase in the likelihood of receiving antipsychotics, ADHD medication, mood stabilisers or antidepressants (Glesener et al., 2018).

#### Service utilisation

Five studies examined the association between psychotropic medication use and utilisation of mental health services. One study reported that young people taking medication were more likely to receive additional services (e.g. case management, social services, school services, outpatient treatment and psychiatric visits) (Brenner et al., 2014). Another study reported that young people with a psychotropic prescription were more likely to visit emergency departments and be hospitalised for mental health concerns (Vish et al., 2024). Two studies reported a significant association between antipsychotic use and inpatient care (Tan et al., 2023) and previous engagement with mental health services prior to prescribing (Rose et al., 2022). Another two studies reported that young people using multiple medications were more likely to engage with speciality mental health services (Raghavan and McMillen, 2008) or outpatient treatment programmes (Brenner et al., 2014).

## Discussion

This review synthesised empirical research on patterns of psychotropic medication use among young people living in out-of-home care. However, the findings were primarily representative of research conducted in the United States and highlighted the lack of international research conducted in other countries. While there is evidence that has revealed increasing psychotropic prescription rates among children and adolescents in other countries such as Australia and New Zealand (Barczyk et al., 2020; Wood et al., 2023), existing research is aggregated and does not provide a breakdown of prevalence rates for those in out-of-home care. To the authors’ knowledge, this review provides the first meta-analysis to investigate the prevalence of psychotropic medication use for this population. The pooled prevalence of psychotropic medication across the included studies was 41.14%, which is greater than international prevalence estimates reported among children and adolescents in the general population (Steinhausen, 2015). The most common medication subclasses were stimulants (and other ADHD medication), antipsychotics and antidepressants, with pooled prevalences of 25.60%, 21.33% and 16.36%, respectively. Estimates for these subclasses were also greater than their prevalence among children and adolescents in the general population, as reported by another recent meta-analysis that calculated the international pooled prevalence for ADHD medication (15.3%), antipsychotics (5.5%) and antidepressants (6.4%) (Piovani et al., 2019).

Similarly, several studies included in this review reported that young people in out-of-home care were prescribed psychotropic medications at disproportionately high rates when compared to young people who were either not living in care or enrolled in other Medicaid eligibility programmes. In particular, young people living in congregate or residential care settings were more likely to be prescribed psychotropic medication than those in other alternative arrangements (e.g. kinship or nonrelative foster care). When considered alongside research highlighting the barriers to accessing mental health services that are experienced by young people living in residential care (Powell et al., 2021), these findings suggest that mental health care in these settings has become deeply entrenched within a medicalised approach that prioritises diagnostic and pharmacological solutions over the provision of accessible, trauma-informed psychosocial support that responds to the lived experiences and complex needs of young people. However, further research in these settings is needed to better understand the factors that influence prescribing patterns in residential care.

The elevated prevalence of psychotropic medication use among young people in out-of-home care may be partially attributed to the complex histories of trauma resulting from exposure to adverse childhood experiences (Bronsard et al., 2016; Engler et al., 2020; Tarren-Sweeney, 2008). However, the findings also suggest that particular demographic characteristics may be associated with an increased likelihood of being prescribed medication, consistent with a recent scoping review by Xu et al. (2023). The increased use of psychotropic medications among boys may be attributed to higher diagnosis rates of certain mental health conditions which are treated with stimulants and antipsychotics (e.g. ADHD, schizophrenia, bipolar disorder), suggesting that sex differences in prescribing patterns may be associated with disproportionate diagnosis rates. Research also suggests that boys in out-of-home care are more likely to display externalising behaviours as a manifestation of early childhood trauma and may be prescribed off-label medications as a form of chemical restraint (Leathers et al., 2021; Linares et al., 2013), while girls are more likely to receive non-pharmacological psychotherapeutic interventions to address underlying mental health needs (Xu et al., 2023).

In addition, greater rates of prescribing have been observed among young people who were White or European American, indicating cultural differences in prescribing patterns. Studies suggest that young people from culturally and linguistically diverse backgrounds are less likely to receive access to mental health services due to a range of societal and systemic barriers, including a lack of culturally sensitive approaches to care (Anderson et al., 2017). While Xu et al. (2023) do not establish a direct relationship between demographic characteristics and prescribing practices in out-of-home care, existing disparities suggest future research is needed. Future research should aim to better understand the factors influencing prescribing decisions and explore how to ensure equitable access to, and appropriate use of, psychotropic medication for young people in care.

Although psychotropic medication can form a key part of a person’s mental health treatment, research indicates that medication may be most effective when prescribed alongside the delivery of non-pharmacological psychotherapy to address underlying trauma and behavioural concerns (Cuijpers et al., 2014). Despite the importance of a holistic approach to mental health treatment, evidence indicates that young people in care who have been prescribed psychotropic medication often do not receive adequate assessments of their mental health and access to ongoing psychotherapy (Keeshin et al., 2020). In particular, there is evidence to suggest that antipsychotics have often been used excessively to manage immediate symptoms, rather than delivered as part of a trauma-informed treatment plan (Palmer et al., 2023). The U.S. Food and Drug Administration has approved the use of first- and second-generation antipsychotics in children for the treatment of schizophrenia and bipolar disorder (Christian et al., 2012), yet several studies indicate that young people in care are more likely to experience off-label prescribing of antipsychotics, without an approved clinical indication for their use (Burcu et al., 2014; Bush et al., 2021; Leckman-Westin et al., 2018; Oerbeck et al., 2021). Given the evidence that the long-term use of antipsychotics in children has been associated with an elevated risk of developing metabolic syndrome and other cardiometabolic abnormalities (Libowitz and Nurmi, 2021), it is important that antipsychotic prescriptions are reviewed periodically, and that young people undergo regular screening to monitor changes in cardiometabolic indicators (e.g. weight, height, blood glucose, lipid levels, etc.) (Mead et al., 2021; Shahidullah et al., 2023).

Despite these concerns, evidence indicates that young people in care have been prescribed antipsychotics and other psychotropic medications as early as preschool age, without ongoing monitoring of effectiveness and safety (dosReis et al., 2014). In a national examination of state-level mechanisms for monitoring the oversight of psychotropic medications in foster care across the United States, Mackie et al. (2017) identified several key components of effective monitoring systems, such as comprehensive database reviews and periodic consultations with mental health experts (e.g. child and adolescent psychiatrists). However, their findings identified interstate variations in the presence of specific monitoring mechanisms and the degree to which they were each relied upon to review the effectiveness and safety of prescriptions for young people in care (Mackie et al., 2017). Furthermore, research has also previously highlighted limitations in how the child welfare and mental health systems communicate and interact with each other to inform effective approaches to prescribing and medication management (Barnett et al., 2016b; Simmel et al., 2021). These limitations are reflective of broader challenges in cross-systems information sharing, including incomplete information on a young person’s history and mental health available to clinicians, a lack of clarity around information sharing policies and uncertainty regarding the specific responsibilities of each professional involved in a young person’s mental health care (Greiner et al., 2019; Hwang et al., 2017). These discrepancies in policy and practice underscore the need for standardised protocols that prioritise the comprehensive assessment of mental health needs and ensure that pharmacological treatment plans are both individualised and developmentally appropriate.

In addition, understanding the complexities of medication management in out-of-home care also requires an approach that recognises the systemic barriers experienced by those directly involved in a young person’s care. For example, caregivers and child welfare staff play a key role in administering medication, monitoring effectiveness and side effects, coordinating clinical appointments and advocating for the young person’s wellbeing. However, caregivers have consistently reported that they are not adequately informed on how to best support young people with managing their medication and have raised concerns about questionable prescribing practices (Barnett et al., 2016a; Barnett et al., 2016b; Bowden et al., 2022; Greiner et al., 2015; Simmel et al., 2021). Similarly, prescribing clinicians have reported that they are not necessarily equipped to respond to the complex histories of trauma and unique mental health needs that are commonly observed among young people living in out-of-home care (Barnett et al., 2016b; Bowden et al., 2022; Simmel et al., 2021).

Evidence also suggests that young people themselves are often not adequately consulted and informed about decisions surrounding their medications. Throughout broader research, young people in out-of-home care have expressed that they are rarely involved in decisions regarding their own health care, and that their mental health needs have been particularly neglected (Smales et al., 2020a; Smales et al., 2020b). Involving young people in decisions regarding their own care has been broadly recognised as essential in providing them with a sense of agency and supporting them to develop skills that will prepare them for the transition into independent living (Caldwell et al., 2019). Within the context of medication management, embracing young people as experts in their own lives can elicit crucial information regarding their mental health symptoms, the necessity and effectiveness of psychotropic medications and the presence of adverse effects (Barnett et al., 2016a; Bowden et al., 2022; Simmel et al., 2021).

### Limitations

The findings of this review must be considered alongside several limitations. First, the findings are largely representative of research conducted in the United States and highlighted the paucity of research from other countries. Given that child welfare policies and medication prescribing procedures may differ between countries, the generalisability of the findings must be interpreted with caution. Second, prevalence data were primarily drawn from administrative or pharmaceutical claims that indicate whether a young person has an existing prescription, which may not necessarily be an accurate indication of actual medication use (Robst et al., 2009). Furthermore, this review was limited to peer-reviewed literature, and the findings were representative of research that has been published over the course of 30 years. As such, this review does not capture grey literature or real-time prevalence data from national or state-wide databases.

During this time, the federal government, state legislators and child welfare agencies in the United States have conducted various inquiries and introduced new policies in response to the overprescribing of psychotropic medications for young people in the child welfare system (Government Accountability Office, 2011; Government Accountability Office, 2017; Mackie et al., 2022). However, the findings do not provide an overall indication of whether these changes have influenced the prevalence of psychotropic medication in out-of-home care. It is also possible that some of the heterogeneity in prevalence observed between studies conducted in different states could be explained by interstate variations in child welfare policies and procedures; however, a comprehensive analysis to disentangle the influence of these contextual differences was beyond the scope of this review.

In addition, qualitative studies that capture the experiences of caregivers, child welfare staff, clinicians and young people themselves were excluded. This gap in the literature limits our understanding of how decisions to prescribe psychotropic medication are made, the challenges that caregivers and clinicians face and the subjective of experiences of young people regarding their treatment (however, a synthesis of this literature is forthcoming).

### Conclusion

This review highlights the complex and elevated use of psychotropic medication among young people in out-of-home care and reveals significant concerns about off-label prescribing and systemic gaps that rely on medical interventions in response to mental health and behavioural needs. These patterns point to broader challenges within care and mental health systems, including inconsistent oversight, limited access to trauma-informed support and gaps in inter-agency collaboration and meaningful involvement of young people. Gaps in the current literature highlight a need for further investment in observational research from countries outside the United States to identify the prevalence of psychotropic medication use and develop a greater understanding of global prescribing patterns for this population.

## Supplemental Material

sj-docx-1-anp-10.1177_00048674251370467 – Supplemental material for Patterns of psychotropic medication use among young people living in out-of-home care: A scoping review and meta-analysis of international literature Supplemental material, sj-docx-1-anp-10.1177_00048674251370467 for Patterns of psychotropic medication use among young people living in out-of-home care: A scoping review and meta-analysis of international literature by Kostas Hatzikiriakidis, Emma Galvin, Luke Patitsas and Helen Skouteris in Australian & New Zealand Journal of Psychiatry

## Notes

1. ‘Out-of-home care’ is henceforth used throughout this article in broadly referring to alternative care arrangements for young people; however, the authors recognise that differences in terminology are observed internationally. Commonly used terms include ‘out-of-home care’ in Australia and ‘looked after children’ in the United Kingdom. In the United States, ‘foster care’ is often used as an umbrella term referring to alternative care arrangements, which includes young people living in either kinship care, with nonrelative caregivers, or in congregate residential care settings. The term ‘out-of-home care’ is used in this article, except in instances where reference is being made to the specific alternative care arrangement reported by a particular study (e.g. nonrelative foster care, kinship care, residential care, group homes, etc.).
