# Supplementary material for: Patterns of psychotropic medication use among young people living in out-of-home care: A scoping review and meta-analysis of international literature
Source: Aust N Z J Psychiatry. 2025 Sep 12;59(11):966–78. doi: 10.1177/00048674251370467 (PMC12569128; doi:10.1177/00048674251370467)
Supplement: sj-docx-1-anp-10.1177_00048674251370467 – Supplemental material for Patterns of psychotropic medication use among young people living in out-of-home care: A scoping review and meta-analysis of international literature [file sj-docx-1-anp-10.1177_00048674251370467.docx]

**Patterns of psychotropic medication use among young people living in out-of-home care: A scoping review and meta-analysis of international literature**

**Supplemental Materials**

| **Table** | **Page Number** |
| --- | --- |
| 1. Search syntax | Page 2 |
| 1. Supplemental Table 1: Study Characteristics | Page 3 |
| 1. Supplemental Table 2: Prevalence and predictors of any medication use | Page 14 |
| 1. Supplemental Table 3: Prevalence and predictors of medication subclasses | Page 17 |
| 1. Supplemental Table 4: Prevalence and predictors of multiple medication use | Page 22 |
| 1. Forest plots synthesising prevalence estimates across five psychotropic subclasses | Page 23 |

**Search Syntax**

The search was conducted using combinations of the terms, "young people" (Population), "out-of-home care" (Context), and "psychotropic medication" (Concept), relevant synonyms, and MeSH headings. The full search syntax for each database has been provided below:

**MEDLINE and EMBASE:**

("young people" OR "young person*" OR "looked after child*" OR "child* of the state" OR adolescen*" OR "teen*" OR "minor" OR "child*" OR "ward* of the state" OR "juvenile*" OR "underage*" OR "youth*") AND ("out-of-home care" OR "out of home care" OR "foster care" OR "kinship care" OR "pre-adoptive home" OR "out of home" OR "group home" OR "congregate care" OR "relative care" OR "living arrangement*" OR "residential care") AND (exp therapeutic uses/ OR "psychotropic*" OR "prescription*" OR "anti?depressant*" OR "anti?psychotic*" OR "stimulant*" OR "anxiolytic*" OR "mood stabili?er* OR "pharmac*" OR "medic*" OR "drug*" OR "medicat*")

**SCOPUS:**

("young people" OR "young person*" OR "looked after child*" OR "child* of the state" OR adolescen*" OR "teen*" OR "minor" OR "child*" OR "ward* of the state" OR "juvenile*" OR "underage*" OR "youth*") AND ("out-of-home care" OR "out of home care" OR "foster care" OR "kinship care" OR "pre-adoptive home" OR "out of home" OR "group home" OR "congregate care" OR "relative care" OR "living arrangement*" OR "residential care") AND ("psychotropic*" OR "prescription*" OR "anti?depressant*" OR "anti?psychotic*" OR "stimulant*" OR "anxiolytic*" OR "mood stabili?er* OR "pharmac*" OR "medic*" OR "drug*" OR "medicat*")

**PsycINFO:**

("young people" OR "young person*" OR "looked after child*" OR "child* of the state" OR adolescen*" OR "teen*" OR "minor" OR "child*" OR "ward* of the state" OR "juvenile*" OR "underage*" OR "youth*") AND ("out-of-home care" OR "out of home care" OR "foster care" OR "kinship care" OR "pre-adoptive home" OR "out of home" OR "group home" OR "congregate care" OR "relative care" OR "living arrangement*" OR "residential care") AND (exp drugs/ OR "psychotropic*" OR "prescription*" OR "anti?depressant*" OR "anti?psychotic*" OR "stimulant*" OR "anxiolytic*" OR "mood stabili?er* OR "pharmac*" OR "medic*" OR "drug*" OR "medicat*")

**CINAHL Plus:**

("young people" OR "young person*" OR "looked after child*" OR "child* of the state" OR adolescen*" OR "teen*" OR "minor" OR "child*" OR "ward* of the state" OR "juvenile*" OR "underage*" OR "youth*") AND ("out-of-home care" OR "out of home care" OR "foster care" OR "kinship care" OR "pre-adoptive home" OR "out of home" OR "group home" OR "congregate care" OR "relative care" OR "living arrangement*" OR "residential care") AND (exp central nervous system agents/ OR "psychotropic*" OR "prescription*" OR "anti?depressant*" OR "anti?psychotic*" OR "stimulant*" OR "anxiolytic*" OR "mood stabili?er* OR "pharmac*" OR "medic*" OR "drug*" OR "medicat*")

Supplemental Table 1. Summary of study characteristics and participant demographics

| **First Author (Year); Country** | **Study Design** | **Data Source/Recruitment** | **Participants and Out-of-Home Care Setting** | **Participant Groups** | **Sample Size** | **Age: N (%) or M (SD)** | **Gender: N (%)** | **Race/Ethnicity: N (%)** | **Mental Health Diagnoses** |
| --- | --- | --- | --- | --- | --- | --- | --- | --- | --- |
| Allaire (2016); United States | Retrospective cohort study (36 US states; 2000-2003) | Medicaid Analytic Extract claim files | Participants: Children in foster care  Criteria: 10-18 years old | Group 1: Children in foster care with an obesity diagnosis | 6,517 | 10 = 6.4%, 11 = 7.2%, 12 = 8.9%, 13 = 10.9%, 14 = 13.2%, 15 = 14.2%, 16 = 15.3%, 17 = 15.1%, 18 = 8.7% | Male = 37.5%  Female = 62.5% | White = 38.5%  Black = 36.2%  Hispanic = 7.9%  Unknown = 14.8%  Other = 2.6% | Not reported |
|  |  |  |  | Group 2: Children in foster care without an obesity diagnosis | 1,255,289 | 10 = 11.2%, 11 = 11.5%, 12 = 11.7%, 13 = 11.7%, 14 = 11.7%, 15 = 11.9%, 16 = 11.8%, 17 = 11.2%, 18 = 7.3% | Male = 51.9%  Female = 48.1% | White = 41.8%  Black = 38.3%  Hispanic = 7.2%  Unknown = 10.7%  Other = 2.0% | Not reported |
| Bozzi (2022); United States | Cross-sectional study (1 US state; 2014) | Data linkage between foster care administrative records, Medicaid medical and pharmacy claims, Kids Count, US Census, and Area Health Resource File | Participants: Children in foster care  Criteria: 5-18 years old | Group 1: Low community adversity | 1,117 | 5-9 = 315 (28%)  10-14 = 345 (31%)  15-18 = 457 (41%) | Male = 542 (49%)  Female = 575 (51%) | White = 559 (50%)  Black = 500 (45%)  Other = 58 (5%) | ADHD = 357 (32%)  Adjustment disorder = 211 (19%)  Anxiety = 307 (27%)  Bipolar/mood disorders = 235 (21%)  Conduct disorder = 289 (26%)  Depression = 94 (8.4%)  PTSD = 148 (13%) |
|  |  |  |  | Group 2: Medium community adversity | 1,389 | 5-9 = 371 (27%)  10-14 = 389 (28%)  15-18 = 629 (45%) | Male = 711 (51%)  Female = 678 (49%) | White = 651 (47%)  Black = 650 (47%)  Other = 88 (6%) | ADHD = 503 (36%)  Adjustment disorder = 289 (21%)  Anxiety = 367 (36%)  Bipolar/mood disorders = 359 (26%)  Conduct disorder = 431 (31%)  Depression = 139 (10%)  PTSD = 253 (18%) |
|  |  |  |  | Group 3: High community adversity | 1,828 | 5-9 = 543 (30%)  10-14 = 518 (28%)  15-18 = 76 (42%) | Male = 890 (49%)  Female = 938 (51%) | White = 200 (11%)  Black = 1,589 (87%)  Other = 39 (2%) | ADHD = 575 (31%)  Adjustment disorder = 343 (19%)  Anxiety = 394 (22%)  Bipolar/mood disorders = 431 (24%)  Conduct disorder = 494 (27%)  Depression = 149 (8.2%)  PTSD = 213 (12%) |
| Breland-Noble (2004); United States | Retrospective cohort study (1 US state; 1999-2001) | Structured interview with foster parents or group home staff | Participants: Youth in community-based residential treatment settings  Criteria: N/A | Group 1: Therapeutic foster care | 184 | M = 14.1 (SD = 2.7) | Male = 48 (26%)  Female = 136 (74%) | White = 55%  African American = 40%  Other = 5% | Not reported |
|  |  |  |  | Group 2: Group homes | 120 | M = 14.3 (SD = 2.2) | Male = 16 (13%)  Female = 104 (87%) | White = 49%  African American = 47%  Other = 4% | Not reported |
| Brenner (2014); United States | Broader randomised controlled trial (1 US state; 2003-2008) | Interviews with treatment foster parents | Participants: Youth in treatment foster care  Criteria: 2-21 years old | Not applicable | 240 | M = 13.0 (SD = 3.8) | Male = 131 (55%)  Female = 109 (55%) | White = 77 (32%)  African American = 138 (58%)  Other = 25 (10%) | Not reported |
| Burcu (2014); United States | Cross-sectional study (1 US state; 2006) | Medicaid administrative claims data | Participants: Medicaid-insured children and adolescents  Criteria: 2-17 years old | Group 1: Foster care | N/R | N/R | N/R | N/R | Not reported |
|  |  |  |  | Group 2: Children’s Health Insurance Program (CHIP) | N/R | N/R | N/R | N/R | Not reported |
|  |  |  |  | Group 3: Temporary Assistance Families (TANF) | N/R | N/R | N/R | N/R | Not reported |
|  |  |  |  | Group 4: Supplemental Security Income (SSI) | N/R | N/R | N/R | N/R | Not reported |
| Bush (2021); United States | Descriptive study (1 US state) | Medicaid administrative data and data from a state-funded team | Participants: Foster children and adolescents  Criteria: 5-19 years old, receiving behavioural and medication review services, combined stimulant and antipsychotic medication | Not applicable | 21 | N/R | Male = 15 (71.4%)  Female = 6 (28.8%) | Caucasian = 8 (38.1%)  African American = 13 (61.9%) | ADHD = 14  Autism spectrum disorder = 4  Learning disorder = 3  Intellectual disability = 2  Expressive language disorder = 1  Mood disorders = 5  Bipolar = 3  Depressive disorder = 2  Dysthymic disorder = 1  Anxiety disorder = 2  Obsessive compulsive disorder = 2  Adjustment disorder = 2  PTSD = 2  Trauma and stress related disorder = 2  Reactive attachment disorder = 1  Relational attachment disorder = 1  Enuresis = 1  Conduct disorder = 3  Intermittent hyperactivity disorder = 2  Intermittent explosive disorder = 1  Oppositional defiant disorder = 5  Impulse control disorder = 1  Borderline personality disorder = 2 |
| Chiang (2024), United States | Cross-sectional study (1 US state, 2015-2020) | Medicaid eligibility file sand fee-for-service and managed care medical encounter claims | Participants: Medicaid-eligible youth  Criteria: 17 years or younger, at least 1 pharmacy claim for psychotropic medication | Group 1: Foster care | 7,369 | Not reported | Not reported | Not reported | Not reported |
|  |  |  |  | Group 2: Low income | 79,064 | Not reported | Not reported | Not reported | Not reported |
|  |  |  |  | Group 3: Children’s Health Program (CHP) | 25,425 | Not reported | Not reported | Not reported | Not reported |
|  |  |  |  | Group 4: Youth with disabilities | 15,114 | Not reported | Not reported | Not reported | Not reported |
| Cosme (2024), United States | Retrospective cohort study (1 US state, 2019-2022) | Electronic medical record system of a single institution partnered with the state’s child welfare agency | Participants: Children in foster care  Criteria: Receiving treatment from a psychiatric institution | Not applicable | 672 | Not reported | Not reported | Not reported | Not reported |
| Crystal (2016); United States | Retrospective cohort study (20 US states, 2005-2010) | Medicaid Analytic eXtract claims-based data | Participants: Medicaid-insured children  Criteria: 0-17 years old | Group 1: Foster care | 2003:  295,053  2010:  279,489 | Not reported | Not reported | Not reported | Not reported |
|  |  |  |  | Group 2: Non-foster care | 2003:  7,228,66  2010:  9,142,407 | Not reported | Not reported | Not reported | Not reported |
| Davis (2021); United States | Cross-sectional study (1 US state; 2012-2017) | Medicaid enrolment and billing claims data | Participants: Children receiving Medicaid services  Criteria: 6-17 years old, treated for high-level polypharmacy, at least one antipsychotic medication claim | Group 1: Foster care | 417 | Mdn = 13.0 (IQR = 11.0-16.0) | Male = 271 (65.0%)  Female = 146 (35.0%) | White, non-Hispanic = 206 (49.4%)  Black, non-Hispanic = 34 (8.2%)  Hispanic = 9 (2.2%)  Other non-Hispanic = 11 (2.6%)  Not provided =157 (37.6%) | Not reported |
|  |  |  |  | Group 2: Not in foster care | 1,823 | Mdn = 12.0 (IQR = 10.0-15.0) | Male = 1,260 (69.1%)  Female = 563 (30.9%) | White, non-Hispanic = 1,246 (68.3%)  Black, non-Hispanic = 129 (7.1%)  Hispanic = 29 (1.6%)  Other non-Hispanic = 41 (2.2%)  Not provided = 378 (20.7%) | Not reported |
| Desjardins (2017); Canada | Cross-sectional study (1 CAN province; 2016) | Questionnaires and semi-structured interviews with children recruited through youth centres | Participants: Children living in foster care group homes and residential rehabilitation centres  Criteria: 6-12 years old | Not applicable | 101 | M = 10, R = 6-12 years | Male = 72 (71.29%)  Female = 30 (28.71%) | Not reported | ADHD = 50 (89.29%)  Learning disorder (10 (17.86%)  Tourette syndrome = 6 (10.71%)  Pervasive developmental disorder (3 (5.36%)  Executive function disorder = 2 (3.57%)  Schizophrenia (traits) = 1 (1.79%)  Bipolarity disorder = 1 (1.79%)  Affective disorder = 2 (3.57%)  Anxiety disorder = 4 (7.14%)  Separation anxiety disorder = 2 (3.57%)  Obsessive compulsive disorder = 2 (3.57%)  Reactive attachment disorder (18 (32.14%)  Relational disorder = 4 (7.14%)  Adjustment disorder = 2 (3.57%)  Oppositional defiant disorder = 12 (21.43%)  Borderline personality disorder = 1 (1.79%)  Dissociative disorder = 1 (1.79%)  Sleep disorder = 1 (1.79%) |
| dosReis (2014); United States | Retrospective cohort study (1 US city; 2009-2011) | Child welfare administrative records and state department of mental health claims data | Participants: Children in foster care  Criteria: Up to the age of 6 years | Not applicable | 1,491 | 0-3 = 1,024 (69%)  4 = 148 (10%)  5 = 173 (12%)  6 = 146 (10%) | Male = 771 (52%)  Female = 720 (48%) | White = 302 (20%)  Black = 1,153 (77%)  Other = 36 (2%) | Disruptive behaviour disorder = 212 (14%)  Internalising disorder = 99 (7%)  Mood disorder = 46 (3%)  Developmental disorder = 23 (2%) |
| dosReis (2011); United States | Cross-sectional study (1 US state; 2003) | Medicaid administrative claims data | Participants: Medicaid-enrolled youth  Criteria: Younger than 20 years old, inpatient or outpatient visit associated with diagnosis, pharmacy claim for an antipsychotic medication | Group 1: Foster care | 2,310 | 0-5 = 6 (0.26%)  5-9 = 270 (11.7%)  10-14 = 895 (38.7%)  15-19 = 1,139 (40.3%) | Male = 1,510 (65.4%)  Female = 800 (34.6%) | White = 1,246 (54.9%)  Black = 864 (37.4%)  Hispanic = 123 (5.3%)  Other = 77 (3.3%) | ADHD = 1,075 (46.5%)  Anxiety = 411 (17.8%)  Conduct disorder = 819 (35.5%)  Depression = 855 (37.0%)  Oppositional defiant disorder = 694 (30.0%)  Substance abuse = 190 (8.2%)  Bipolar disorder = 469 (20.3%)  Psychoses = 366 (15.8%)  Schizophrenia = 161 (6.9%)  Autism = 36 (1.6%) |
|  |  |  |  | Group 2: Supplemental Security Income (SSI) | 8,787 | 0-5 = 51 (0.58%)  5-9 = 1,750 (19.9%)  10-14 = 4,061 (46.2%)  15-19 = 2,925 (33.3%) | Male = 6,551 (74.6%)  Female = 2,236 (25.5%) | White = 6,238 (70.9%)  Black = 1,479 (16.8%)  Hispanic = 308 (3.5%)  Other = 762 (8.8%) | ADHD = 4,925 (56.1%)  Anxiety = 968 (11.0%)  Conduct disorder = 2,058 (23.4%)  Depression = 2,557 (29.1%)  Oppositional defiant disorder = 2,224 (25.3%)  Substance abuse = 271 (3.1%)  Bipolar disorder = 1,949 (22.2%)  Psychoses = 1,309 (14.9%)  Schizophrenia = 454 (5.2%)  Autism =803 (9.1%) |
|  |  |  |  | Group 3: Temporary Assistance Families (TANF) | 3,631 | 0-5 = 45 (1.2%)  5-9 = 1,005 (27.7%)  10-14 = 1,622 (44.7%)  15-19 = 959 (26.4%) | Male = 2,318 (63.8%)  Female = 1,313 (36.2%) | White = 2,477 (68.2%)  Black = 567 (15.6%)  Hispanic = 145 (3.9%)  Other = 442 (12.2%) | ADHD = 18,98 (52.3%)  Anxiety = 470 (12.9%)  Conduct disorder = 785 (21.6%)  Depression = 1,385 (38.1%)  Oppositional defiant disorder = 889 (24.5%)  Substance abuse = 153 (4.2%)  Bipolar disorder = 550 (15.2%)  Psychoses = 479 (13.2%)  Schizophrenia = 134 (3.7%)  Autism = 33 (0.91%) |
| dosReis (2001); United States | Cross-sectional study (1 US state; 1996) | Medicaid administrative claims data | Participants: Medicaid-enrolled youth  Criteria: Younger than 20 years old, at least one medical or prescription claim associated with a mental health disorder or psychotherapeutic medication | Group 1: Foster care | 310 | 0-5 = 93 (30%)  6-14 = 133 (43%)  15-19 = 84 (27%) | Male = 156 (50%)  Female = 154 (50%) | White = 110 (35%)  African American = 142 (46%)  Other = 58 (19%) | Adjustment disorder = 64 (21%)  ADHD = 48 (16%)  Depression = 46 (15%)  Developmental disorder = 32 (10%)  Conduct disorder = 26 (8%)  Oppositional defiant disorder = 24 (8%)  Anxiety = 20 (7%)  Substance abuse = 15 (5%)  Learning disorder = 12 (4%)  Psychoses = 5 (2%) |
|  |  |  |  | Group 2: Supplemental Security Income (SSI) | 775 | 0-5 = 232 (30%)  6-14 = 380 (49%)  15-19 = 163 (21%) | Male = 281 (36%)  Female = 494 (64%) | White = 372 (48%)  African American = 143 (18%)  Other = 260 (34%) | Adjustment disorder = 18 (2%)  ADHD = 55 (7%)  Depression = 53 (7%)  Developmental disorder = 56 (7%)  Conduct disorder = 23 (3%)  Oppositional defiant disorder = 15 (2%)  Anxiety = 13 (2%)  Substance abuse = 15 (2%)  Learning disorder = 37 (5%)  Psychoses = 26 (3%) |
|  |  |  |  | Group 3: Other Aid | 14,422 | 0-5 = 7,932 (55%)  6-14 = 5,336 (37%)  15-19 = 1,154 (8%) | Male = 5,065 (35%)  Female = 9,357 (65%) | White = 3,180 (22%)  African American = 6,223 (43%)  Other = 5,019 (35%) | Adjustment disorder = 86 (0.6%)  ADHD = 121 (0.8%)  Depression = 102 (0.7%)  Developmental disorder = 149 (1.0%)  Conduct disorder = 51 (0.4%)  Oppositional defiant disorder = 26 (0.2%)  Anxiety = 31 (0.2%)  Substance abuse = 55 (0.4%)  Learning disorder = 62 (0.4%)  Psychoses = 16 (0.1%) |
| Ferguson (2006); United States | Retrospective chart review study (1 US state; 2000) | Medical assistance pharmaceutical records | Participants: Children in foster care  Criteria: 18 years or younger, not in foster care for medical reasons or in emergency care | Not applicable | 473 | Av. = 11 years  0-2 = 44 (9.3%)  3-5 = 48 (10.1%)  6-8 = 49 (10.4%)  9-11 = 56 (11.8%)  12-14 = 123 (26.0%)  15-17 (32.3%) | Male = 253 (53%)  Female = 220 (47%) | European American = 303 (60%)  American Indian = 170 (33%)  African American = 31 (6%)  Other or not identified = 5 (1%) | Not reported |
| Fontanella (2015); United States | Retrospective cohort study (1 US state; 2007-2010) | Medicaid claims, Area Resource File, and the Ohio State Psychology and Social Work Licensure Boards | Participants: Youth in foster care  Criteria: 5-17 years old, at least two claims for serious emotional disturbances (e.g., schizophrenia, psychosis, bipolar) | Not applicable | 952 | 5-9 = 311 (32.7%)  10-17 = 641 (67.3%) | Male = 461 (48.4%)  Female = 491 (51.6%) | White = 566 (59.5%)  Non-White = 386 (40.6%) | ADHD = 244 (25.6%)  Schizophrenia/psychosis = 61 (6.4%)  Bipolar = 891 (93.6%)  Anxiety = 191 (20.1%)  Conduct disorder = 238 (25.0%)  Substance abuse = 52 (5.5%) |
| Fontanella (2014); United States | Cross-sectional study; (1 US state; 2002-2008) | Medicaid fee-for-service claims data | Participants: Medicaid-enrolled youth  Criteria: 17 years or younger, with at least 1 prescription claim for psychotropic medication | Group 1: Foster care | 4,906 | 0-6 = 262 (5.3%)  7-12 = 2,154 (43.9%)  13-17 = 2,490 (50.7%) | Male = 3,192 (65.1%)  Female = 1,714 (34.9%) | White = 3,029 (61.7%)  Black = 1,806 (36.8%)  Other = 71 (1.4%) | ADHD = 1,990 (40.6%)  Adjustment disorder = 243 (4.9%)  Anxiety = 307 (6.3%)  Autism = 23 (0.5%)  Disruptive disorder = 557 (11.3%)  Mood disorders = 570 (11.6%)  Mental retardation = 84 (1.7%)  Schizophrenia = 31 (0.6%)  Other = 558 (11.4%) |
|  |  |  |  | Group 2: Disability | 8,592 | 0-6 = 770 (9.0%)  7-12 = 4,151 (48.3%)  13-17 = 3,671 (42.7%) | Male = 6,182 (72.0%)  Female = 2,410 (28.0%) | White = 6,238 (72.6%)  Black = 2,221 (25.8%)  Other = 133 (1.5%) | ADHD = 2,331 (27.1%)  Adjustment disorder = 116 (1.3%)  Anxiety = 159 (1.8%)  Autism = 342 (4.0%)  Disruptive disorder = 496 (5.8%)  Mood disorders = 623 (7.2%)  Mental retardation = 1,155 (13.4%)  Schizophrenia = 47 (0.5%)  Other = 1,218 (14.2%) |
|  |  |  |  | Group 3: Low income | 36,813 | 0-6 = 2,979 (8.1%)  7-12 = 20,701 (56.2%)  13-17 = 13,133 (35.7%) | Male = 24,825 (67.4%)  Female = 11,988 (32.6%) | White = 30,353 (82.4%)  Black = 6,111 (16.6%)  Other = 349 (0.9%) | ADHD = 20,299 (55.1%)  Adjustment disorder = 142 (3.9%)  Anxiety = 1,151 (3.1%)  Autism = 159 (0.4%)  Disruptive disorder = 3,023 (8.2%)  Mood disorders = 3,317 (9.0%)  Mental retardation = 246 (0.7%)  Schizophrenia = 105 (0.3%)  Other = 2,919 (7.9%) |
| Gardner (2016); United States | Retrospective cohort study (1 US state; 2003-2014) | Private health information database and Medicaid records | Participants: Teenage girls in foster care  Criteria: 15-18 years old | Group 1: Foster care | 743 | Not reported | Male = 0 (0%)  Female = 743 (100%) | Not reported | Not reported |
|  |  |  |  | Group 2: Non-foster Medicaid | 2,636 | Not reported | Male = 0 (0%)  Female = 2,636 (100%) | Not reported | Not reported |
|  |  |  |  | Group 3: Private coverage | 1,947 | Not reported | Male = 0 (0%)  Female = 1,947 (100%) | Not reported | Not reported |
| Glesener (2018); United States | Cross-sectional (1 US state; 2012) | Medical assistance records, Social Services records, and Medicaid database | Participants: Children in foster care  Criteria: 17 years or younger, no developmental disability, not living in residential or shelter facilities | Group 1: European American | 332 | 0-4 = 126 (38.0%)  5-9 = 75 (22.6%)  10-14 = 71 (21.4%)  15-17 = 60 (18.1%) | Male = 172 (51.8%)  Female = 160 (48.2%) | European American = 332 (53%) | Not reported |
|  |  |  |  | Group 2: American Indian | 223 | 0-4 = 91 (40.8%)  5-9 = 52 (23.3%)  10-14 = 37 (16.6%)  15-17 = 43 (19.3%) | Male = 128 (57.4%)  Female = 95 (42.6%) | American Indian = 223 (36%) | Not reported |
|  |  |  |  | Group 3: African American | 71 | 0-4 = 32 (45.0%)  5-9 = 17 (23.9%)  10-14 = 14 (19.7%)  15-17 = 8 (11.3%) | Male = 43 (60.4%)  Female = 28 (39.4%) | African American = 71 (11%) | Not reported |
| Green (2005); United States | Cross-sectional study (1 US state; 2001) | Case file reviews and structured interviews with case managers | Participants: Children in foster care  Criteria: N/A | Not applicable | 722 | 0-5 = 168 (23.3%)  6-12 = 249 (34.5%)  13-18 = 257 (35.6%)  19+ = 39 (5.4%) | Male = 389 (53.9%)  Female = 333 (46.1%) | White = 381 (52.8%)  Black = 277 (38.4%)  Hispanic = 33 (4.6%)  Asian = 11 (1.5%)  Other = 21 (2.9%) | ADHD = 31 (20%)  Bipolar disorder = 14 (9%)  Major depression = 12 (8%)  Dysthymia = 5 (3%)  PTSD = 15 (10%)  Adjustment disorder (emotion) = 4 (3%)  Adjustment disorder (anxiety) = 5 (3%)  Adjustment disorder (depression) = 1 (<1%)  Conduct disorder = 4 (3%)  Intermittent explosive disorder = 5 (3%)  Oppositional defiant disorder = 2 (1%) |
| Keast (2019); United States | Cross-sectional study (1 US state; 2016) | Medicaid claims data and foster care eligibility data | Participants: Medicaid-eligible youth  Criteria: 0-20 years | Group 1: Foster care | 9,325 | 0-4 = 4,090 (43.9%)  5-12 = 3,802 (40.8%)  13-20 = 1,433 (15.4%) | Male = 4,857 (52.1%)  Female = 4,468 (47.9%) | White = 5,150 (55.2%)  Black = 1,122 (12.0%)  Asian = 15 (0.2%)  American Indian or Alaskan Native = 904 (9.7%)  Native Hawaiian or Pacific Islander = <10 (0.1%)  Mixed = 2,124 (22.8%)  Unknown = <10 (0.0%) | Not reported |
|  |  |  |  | Group 2: Not in foster care | 639,868 | 0-4 = 186,691 (29.2%)  5-12 = 266,011 (41.6%)  13-20 = 187,166 (29.3%) | Male = 323,229 (50.5%)  Female = 316,639 (49.5%) | White = 381,237 (59.6%)  Black = 71,532 (11.2%)  Asian = 9,542 (1.5%)  American Indian or Alaskan Native = 73,475 (11.5%)  Native Hawaiian or Pacific Islander = 2,420 (0.4%)  Mixed = 101,592 (15.9%)  Unknown = 70 (0.1%) | Not reported |
| Leathers (2021); United States | Cross-sectional study (1 US state; year not reported) | Telephone interviews with foster parents | Participants: Children in foster care  Criteria: 8-14 years old, under state guardianship, history of moves or prior hospitalisation | Group 1: Foster children | 167 | R = 8-14 years | Male = 89 (62.6%)  Female = 55 (37.4%) | African American = 61.2%  White = 25.2%  Mixed race = 9.4%  Asian = 2.2%  Other = 2.2%  Latino = 11.5% | ADHD = 79 (52.2%)  Autism = 4 (2.8%)  Schizophrenia = 3 (2.1%)  Bipolar disorder = 13 (9.0%)  Depression = 14 (9.8%)  Anxiety = 4 (2.8%)  PTSD = 9 (6.3%)  Disruptive behaviour disorder 29 (20%) |
|  |  |  |  | Group 2: Foster parents | 144 | M = 51.67 (SD = 11.36) | Male = 10.8%  Female = 89.2% | African American = 64.7%  White = 32.4%  Other = 2.9%  Latino = 1.4% | Not applicable |
| Leckman-Westin (2018); United States | Cross-sectional study (10 US states; 2008) | Medicaid Analytic Extract data files | Participants: Medicaid beneficiaries  Criteria: 0-21 years old, at least 1 antipsychotic claim or encounter | Group 1: Foster care | 31,375 | 0-5 = 792 (2.5%)  6-11 = 9,000 (28.7%)  12-17 = 18,521 (59.0%)  18-20 = 3,062 (9.8%) | Male = 20,088 (64%)  Female = 11,287 (36%) | Hispanic any race = 2,838 (9.0%)  White non-Hispanic = 18,879 (60.2%)  Black non-Hispanic = 8,447 (26.9%)  Other = 439 (1.4%)  Unknown = 772 (2.5%) | Not reported |
|  |  |  |  | Group 2: Supplemental Security Income (SSI) | 47,694 | 0-5 = 1,290 (2.7%)  6-11 = 13,722 (28.8%)  12-17 = 21,424 (44.9%)  18-20 = 11,258 (23.6%) | Male = 35,369 (74.2%)  Female = 12,325 (25.8%) | Hispanic any race = 5,321 (11.2%)  White non-Hispanic = 21,249 (44.6%)  Black non-Hispanic = 7,891 (16.5%)  Other = 1,257 (2.6%)  Unknown = 11,976 (25.1%) | Not reported |
|  |  |  |  | Group 3: Other Aid | 6,289 | 0-5 = 167 (2.7%)  6-11 = 1,452 (23.1%)  12-17 = 2,040 (32.4%)  18-20 = 2,630 (41.8%) | Male = 3,984 (63.3%)  Female = 2,305 (36.7%) | Hispanic any race = 1,152 (18.3%)  White non-Hispanic = 3,805 (60.5%)  Black non-Hispanic = 899 (14.3%)  Other = 208 (3.3%)  Unknown = 225 (3.6%) | Not reported |
|  |  |  |  | Group 4: Income Aid | 58,842 | 0-5 = 2,338 (4.0%)  6-11 = 22,126 (37.6%)  12-17 = 29,088 (49.4%)  18-20 = 5,290 (9.0%) | Male = 39,270 (66.7%)  Female = 19,572 (33.3%) | Hispanic any race = 5,955 (10.1%)  White non-Hispanic = 41,814 (71.1%)  Black non-Hispanic = 8,694 (14.8%)  Other = 969 (1.6%)  Unknown = 1,410 (2.4%) | Not reported |
| Linares (2013); United States | Cross-sectional study; (1 US state, 2002-2007) | Yearly interview assessments with biological and foster parents of children drawn from foster care agencies | Participants: Children in foster care  Criteria: 3-14 years old, not in kinship, no developmental disability | Not applicable | 252 | 3.0-7.9 = 143 (57%)  8.0-11.9 = 85 (34%)  12+ = 24 (9%) | Male = 141 (56%)  Female = 111 (44%) | African American = 118 (46%)  Latino = 66 (26%)  Mixed/Other (Caucasian, Asian) = 68 (27%) | ADHD = 138 (55%)  Oppositional defiant = 100 (40%)  Conduct = 63 (25%)  Separation anxiety = 117 (46%)  Generalised anxiety = 20 (8%)  Major depression = 29 (12%)  Elimination = 54 (21%) |
| McLeigh (2023); United States | Retrospective chart review (1 US state, 2017-2020) | Data collected from integrated primary care clinics for children in foster care | Participants: Children in foster care  Criteria: 3-20 years old | Not applicable | 3,067 | 3-5 = 1,072 (35.0%)  6-11 = 1,225 (39.9%)  12-20 = 770 (25.1%) | Male = 1,598 (52.1%)  Female = 1,469 (47.9%) | White or Caucasian = 829 (27.0%)  Black or African American = 971 (31.7%)  Hispanic = 647 (21.1%)  Other = 41 (1.3%)  Unknown = 578 (18.9%) | ADHD = 692 (22.6%)  Bipolar = 37 (1.2%)  Depression = 413 (13.5%)  Anxiety = 295 (9.6%)  Obsessive compulsive and related = 38 (1.2%)  Trauma and stressor related disorders = 967 (31.5%)  Disruptive, impulse control, and conflict disorders = 314 (10.2%)  Personality disorders = 1 (0%)  Dissociative disorders = 0 (0%)  Somatic disorders = 9 (0.3%)  Gender dysphoria = 5 (0.2%)  Substance-related and addictive disorders = 60 (2.0%) |
| McMillen (2004); United States | Retrospective cohort study (1 US state; 2001-2003) | Interviews with youth in their residences | Participants: Youth in foster care  Criteria: 17 years old, IQ above 70 | Not applicable | 406 | M = 16.99 (SD = 0.09) | Male = 178 (44%)  Female = 228 (56%) | White = 178 (44%)  African American = 206 (51%)  Mixed race = 15 (4%)  American Indian = 3 (1%)  Asian = 2 (<1%)  Latino = 1 (<1%)  Middle Eastern = 1 (<1%) | Psychiatric disorder in the past year = 149 (37%)  Lifetime psychiatric disorders = 246 (61%) |
| Monson (2023); United States | Retrospective drug review (1 US state, 2019-2022) | Data collected as part of routine monitoring by foster care nurse case managers, collected from prescribers and foster families | Participants: Youth in foster care  Criteria: 0-18 years old | Group 1: Utah Psychotropic Oversight Program (antipsychotics) | 757 | 0-6 = 21 (3%)  7-12 = 178 (24%)  13+ = 556 (74%) | Male = 436 (58%)  Female = 319 (42%) | White = 670 (89%)  American Indian/Alaskan Native = 26 (3%)  Asian = 2 (0%)  Black/African American = 43 (6%)  Multi racial/other = 7 (1%)  Native Hawaiian/Other Pacific = 5 (1%)  Unknown = 2 (0%)  Hispanic = 142 (19%)  Non-Hispanic = 605 (80%) | Not reported |
|  |  |  |  | Group 2: Utah Psychotropic Oversight Program (no antipsychotics) | 1,004 | 0-6 = 296 (29%)  7-12 = 258 (26%)  13+ =452 (45%) | Male = 520 (52%)  Female =486 (48%) | White = 885 (88%)  American Indian/Alaskan Native = 25 (2%)  Asian = 8 (1%)  Black/African American = 55 (5%)  Multi racial/other = 17 (2%)  Native Hawaiian/Other Pacific = 11 (1%)  Unknown = 5 (0%)  Hispanic = 201 (20%)  Non-Hispanic = 792 (79%) | Not reported |
|  |  |  |  | Group 3: Foster youth not in Utah Psychotropic Oversight Program | 6,762 | 0-6 = 4,053 (60%)  7-12 = 1,670 (25%)  13+ =1,039 (15%) | Male = 3,453 (51%)  Female =3,309 (49%) | White = 5,890 (87%)  American Indian/Alaskan Native = 215 (3%)  Asian = 32 (0%)  Black/African American = 257 (4%)  Multi racial/other = 128 (2%)  Native Hawaiian/Other Pacific = 118 (2%)  Unknown = 112 (2%) | Not reported |
| Narendorf (2011); United States | Cross-sectional study (1 US state; 2009) | Child welfare records and interviews with youth and key informants | Participants: Youth in treatment foster care  Criteria: 16-18 years, IQ above 70, receiving psychiatric medication or a history of psychiatric hospitalisation | Not applicable | 8 | Av. = 17.4 | Male = 2 (25%)  Female = 6 (75%) | African American = 6 (75%)  Caucasian = 1 (12.5%)  Biracial = 1 (12.5%) | ADHD = 1 (12.5%)  Mood disorder = 2 (25%)  PTSD = 2 (25%)  Oppositional defiant disorder = 4 (50%)  Conduct disorder = 1 (12.5%) |
| Nunes (2022); United States | Retrospective cohort study (1 US state; 2011-2020) | California Child Welfare Indicators Project (Medicaid claims and individual foster care data | Participants: Youth in foster care  Criteria: N/A | Time 1: 2011 | 78,231 | 0-5 = 31,452  6-10 = 14,782  11-15 = 19,697  16-17 = 12,426 | Male = 41,055  Female = 37,291 | Black = 18,970  White = 1,615  Latino = 1,677  Asian/Pacific Islander = 83  Native American = 80 | Not reported |
|  |  |  |  | Time 2: 2020 | 68,386 | 0-5 = 30,849  6-10 = 14,852  11-15 = 15,150  16-17 = 7,546 | Male = 35,039 (49.1%)  Female = 33,349 (43.3%) | Black = 13,448 (19.7%)  White = 14,807 (21.7%)  Latino = 36,959 (54.0%)  Asian/Pacific Islander = 1,361 (2.0%)  Native American = 832 (1.2%) | Not reported |
| Oerbeck (2020); Norway | Cross-sectional study (national; 2016) | Norwegian Prescription Database (norPD) | Participants: Children in residential care institutions  Criteria: 0-20 years old | Not applicable | 2,171 | M = 14 (82% were 13+) | Male = 60%  Female = 40% | Not reported | Not reported |
| Oerbeck (2021); Norway | Cross-sectional study (national; 2016) | Norwegian Prescription Database (norPD) | Participants: Children in residential care institutions  Criteria: 0-20 years | Not applicable | 1,856 | M = 14 (81% were 13+) | Male = 54%  Female = 46% | Not reported | Not reported |
| Palmer (2023); United States | Retrospective cohort study (1 US state; 2009-2016) | Linked administrative Medicaid and child protective data | Participants: Early adolescents in foster care  Criteria: 10-13 years old | Not applicable | 2,998 | 10 = 27.79%  11 = 22.98%  12 = 24.78%  13 = 24.45% | Male = 49.25%  Female = 50.65% | White = 41.19%  Black = 26.55%  American Indian = 3.24%  Asian or Pacific Islander = 1.00%  Multiple races non-Hispanic = 12.51%  Hispanic (any race) = 15.51% | Not reported |
| Park (2019); United States | Prospective cohort study (1 US state; 2012-2015) | State administrative child welfare data from the California Youth Transitions to Adulthood Study | Participants: Foster youth  Criteria: Transition age (16.75-17.75 years old) | Not applicable | 611 | Not applicable (17 in 2013 and 19 in 2015) | Male = 243 (40%)  Female = 368 (60%) | White = 139 (17.6%)  Black = 90 (17.3%)  Hispanic = 270 (47.4%)  Mixed race = 90 (15.2%)  Other = 21 (2.4%) | Psychotic thinking = 48 (8.0%)  Mania = 84 (13.6%)  Major depressive episode = 134 (21.5%)  Social phobia = 33 (5.1%)  PTSD = 48 (7.2%)  Alcohol abuse/dependence = 89 (12.7%)  Drug abuse/dependence = 144 (22.7%) |
| Raghavan (2010); United States | Cross-sectional study (8 US states; 2002-2004) | National Survey of Child and Adolescents Wellbeing (NSCAW) | Participants: Children in the child welfare system  Criteria: 0-14 years old | Not applicable | 3,017 | M = 10.2, R = 3-18 years | Male = 49.9%  Female = 50.1% | White = 48%  African American = 27.2%  Hispanic = 18.7%  Other = 6.1% | Not reported |
| Raghavan (2008); United States | Cross-sectional study (1 US state; 2001-2003) | Interviews with adolescents leaving foster care | Participants: Foster youth  Criteria: Transition age (17 years old) | Not applicable | 403 | 17 years = 403 (100%) | Male = 177 (44%)  Female = 226 (56%) | White = 177 (43.9%)  Black = 206 (51.1%)  Hispanic = 1 (0.2%)  Other = 19 (4.7%) | ADHD = 44 (10.9%)  Manic episode = 21 (5.2%)  Major depressive disorder = 71 (17.6%)  PTSD = 28 (6.9%)  Disruptive behaviour disorder = 63 (15.6%)  Substance use disorder = 72 (17.9%) |
| Robst (2009); United States | Retrospective cohort study (1 US state; 2003-2006) | Medicaid claims data | Participants: Medicaid-enrolled youth in out-of-home care  Criteria: N/A | Group 1: Therapeutic foster care | 860 | 0-5 = 49 (20.2%)  6-12 = 796 (32.8%)  13-18 = 1,584 (65.2%) | Male = 1,386 (57.1%)  Female = 1,043 (42.9%) | White = 1,190 (48.99%)  Black = 705 (29.02%)  Other = 534 (21.98%) | ADHD = 577 (23.8%)  Schizophrenia & psychosis = 100 (4.1%)  Mood/affective disorders = 764 (31.5%)  Anxiety disorders = 349 (14.4%)  Adjustment disorders = 118 (4.9%)  Disruptive behaviour disorders = 457 (18.8%)  Other = 64 (2.6%) |
|  |  |  |  | Group 2: Statewide Inpatient Psychiatric Program (SIPP) | 1,080 |  |  |  |  |
|  |  |  |  | Group 3: Therapeutic Group Care (TGC) | 489 |  |  |  |  |
| Rose (2022); United States | Retrospective cohort study (1 US state; 2011-2018) | Medicaid claims data | Participants: Youth in foster care  Criteria: 5-18 years old, diagnosis of disruptive behaviour disorder or one of two conditions indicated for antipsychotic medication | Not applicable | 10,397 | Not reported | Male = 5,844 (57%)  Female = 4,553 (43%) | Latino/Latina/Hispanic = 457 (5%)  Black or African American = 4,463 (44%)  Asian/Pacific Islander = 31 (0%)  American Indian/Native American = 262 (3%) | ADHD = 4,014 (39%)  Learning disabled -intellectual/developmental disability = 1,601 (15%)  Psychosis = 891 (9%)  Bipolar = 1,121 (11%)  Depression = 999 (10%)  Anxiety disorder = 1,725 (17%)  Adjustment disorder = 2,510 (24%)  PTSD = 969 (9%)  Disruptive behaviour disorder = 9,700 (93%) |
| Rose (2023); United States | Retrospective cohort study (1 US state; 2011-2018) | Medicaid claims data | Participants: Youth in foster care  Criteria: 5-15 years old, ADHD with subsequent diagnosis of disruptive behaviour disorder | Not applicable | 10,005 | Av. = 8 years 10 months (SD = 3 years) | Male = 60%  Female = 40% | Hispanic = 440 (4.4%)  Black/African American = 3,499 (35%)  White = 5,738 (57%)  American Indian or Native American = 182 (2%  Asian or Pacific Islander = 25 (<1%) | ADHD = 10,005 (100%)  Learning or intellectual/developmental disability = 1,044 (10.4%)  Depression = 303 (3.0%)  Anxiety disorder = 450 (4.5%)  Disruptive behaviour disorder = 35% |
| Rubin (2012); United States | Cross-sectional study (50 US states; 2002-2007) | Medicaid Analytic Extract data files | Participants: Youth in foster care  Criteria: 3-18 years old | Time 1: 2002 | 686,080 (average of eligible foster youth in any given year) | 3-5 = 15%  6-11 = 34%  12-18 = 51% | Male = 52%  Female = 48% | White = 48%  Black = 37%  Hispanic = 11%  Other = 4% | Not reported |
|  |  |  |  | Time 2: 2007 |  |  |  |  | Not reported |
| Rubin (2009); United States | Cross-sectional study (50 US states; 2001) | Medicaid Analytic Extract data files | Participants: Medicaid-enrolled youth  Criteria: 3-18 years old, autism spectrum disorder | Group 1: Foster care | 43,306 | 3-5 = 14.4%  6-11 = 45.2%  12-17 = 40.4% | Male = 73.2%  Female = 26.8% | White = 51.6%  Black = 32.8%  Hispanic = 4.6%  Other = 11.0% | ADD = 36.4%  Schizophrenia = 3.0%  Bipolar = 7.9%  Depression = 11.3%  Anxiety disorder = 3.6%  Conduct disorder = 28.6%  Mental retardation = 20.8% |
|  |  |  |  | Group 2: Supplemental Security Income (SSI) |  | 3-5 = 15.6%  6-11 = 52.1%  12-17 = 32.3% | Male = 78.8%  Female = 21.2% | White = 48.7%  Black = 19.0%  Hispanic = 5.8%  Other = 26.6% | ADD = 20.0%  Schizophrenia = 1.0%  Bipolar = 2.9%  Depression = 3.2%  Anxiety disorder = 2.0%  Conduct disorder = 12.3%  Mental retardation = 23.7% |
| Scorzzaro (2015); United States | Cross-sectional study (1 US state) | Case files of foster youth drawn from a county databank | Participants: Children in foster care  Criteria: N/A | Not applicable | 128 | Not reported | Male = 52.3%  Female = 47.7% | African American = 74 (58%)  White = 36 (28%)  Hispanic = 3 (2%)  Other = 16 (13%) | Not reported |
| Solerdelcoll (2024); Spain | Retrospective observational study (1 US state, 2014-2017) | Computerised clinical charts and patient records | Participants: Youth in foster care admitted to an inpatient psychiatric ward  Criteria: Under 18 years old | Group 1: Youth in foster care in an inpatient psychiatric ward | 89 | M = 14.5 (SD = 2.2) | Male = 37 (41.6%)  Female = 52 (58.4%) | White = 49 (55.1%) | Any psychotic disorder = 24 (27.0%)  Any mood disorder = 37 (41.6%)  ADHD = 20 (13.4%)  ODD = 17 (9.1%)  Conduct disorder = 70 (78.7%)  Autism spectrum disorder = 10 (11.2%)  Any eating disorder = 5 (5.6%)  OCD = 1 (1.1%)  Intellectual disability = 27 (30.3%)  PTSD = 5 (5.6%)  Tourette syndrome = 2 (2.3%)  Any substance use disorder = 44 (49.4%) |
|  |  |  |  | Group 2: Non-foster youth living at home | 247 | M = 15.2 (SD = 1.8) | Male = 95 (38.5%)  Female = 152 (61.5%) | White = 205 (82.3%) | Any psychotic disorder = 47 (19.0%)  Any mood disorder = 94 (38.1%)  ADHD = 33 (22.5%)  ODD = 11 (4.5%)  Conduct disorder = 36 (14.6%)  Autism spectrum disorder = 33 (13.4%)  Any eating disorder = 63 (25.5%)  OCD = 14 (5.7%)  Intellectual disability = 16 (6.5%)  PTSD = 5 (2.0%)  Tourette syndrome = 5 (2.0%)  Any substance use disorder = 68 (27.5%) |
| Soto-Ramirez (2024); United States | Retrospective cohort study (1 US state (2018-2019) | Foster care services data, medical claims; medical claims, Medicaid, and pharmacy data; and mental health data | Participants: Children in foster care  Criteria: 2-17 years old | Not applicable | 1,002 | Average age of entry = 9 (R = 2-17) | Male = 523 (52.2%)  Female = 479 (47.8%) | White/Caucasian = 475 (47.74%)  Black/African American = 334 (33.57%)  Hispanic = 75 (7.54%)  More than one race = 26 (2.61%)  Other/unknown = 85 (8.54%) | PTSD = 67 (6.69%)  Conduct disorder = 103 (10.28%)  Depression or anxiety disorders = 83 (8.28%)  Adjustment disorder = 256 (25.55%)  ADHD = 164 (16.37%)  Other = 112 (11.18%) |
| Spence (2019); United States | Retrospective cohort study (1 US state; 2010-2015) | Administrative Medicaid mental health and pharmacy claims data | Participants: Children in foster care  Criteria: 21 years or younger, initiated antipsychotic medication | Not applicable | 753 | M = 15.1 (SD = 4.4) | Male = 384 (51%)  Female = 369 (49%) | African American = 519 (69%) | Not reported |
| Tai (2016); United States | Retrospective cohort study (1 US state; 2010-2013) | Child welfare administrative records and Medicaid claims data | Participants: Youth in foster care  Criteria: 21 years or younger, initiated antipsychotic prescription | Not applicable | 390 | M = 14.6 (SD = 3.9)  < 5 = 13 (3.3%)  6-10 = 48 (12.3%)  11-15 = 129 (33.1%)  16-21 = 200 (51.3%) | Male = 228 (58.5%)  Female = 162 (41.5%) | White = 69 (17.7%)  Black/African American = 312 (80.0%)  Others = 9 (2.3%) | Externalising disorders = 390 (100%)  Internalising disorders = 145 (37.2%)  Developmental disorders = 15 (3.8%) |
| Tan (2023); United States | Retrospective cohort study (1 US state; 2012-2017) | Electronic health record data linked to administrative child welfare data | Participants: Youth in foster care  Criteria: 10 years or older | Group 1: Foster care | 2,758 | M = 14.71 (SD = 3.14) | Male = 1402 (50.83%)  Female = 1,356 (49.17%) | Persons of colour = 947 (34.34%) | ADHD = 17.3%  Stress-related disorder = 3.1%  Disruptive behaviour disorder = 7.2%  Conduct disorder = 2.3%  Substance use disorder = 1.6%  Other mental health diagnosis = 12.8% |
|  |  |  |  | Group 2: Comparison youth not in foster care | 2,787 | M = 15.50 (SD = 3.80) | Male = 1,412 (50.66%)  Female = 1,375 (49.33%) | Persons of colour = 1,004 (36.02%) | ADHD = 6.5%  Stress-related disorder = 0.5%  Disruptive behaviour disorder = 2.5%  Conduct disorder = 0.5%  Substance use disorder = 0.6%  Other mental health diagnosis = 7.8% |
| Vanderwerker (2014); United States | Retrospective cohort study (44 US states; 2009) | Medicaid Analytic Extract claims data | Participants: Medicaid enrolled youth  Criteria: N/A | Group 1: Foster care | 301,894 | 0-5 = 18.1%  6-9 = 22.7%  10-13 = 25.6%  14-18 = 33.6% | Male = 51.6%  Female = 48.4% | White = 46.8%  Black/African American = 36.8%  Hispanic = 12.9%  Other = 3.5% | Not reported |
|  |  |  |  | Group 2: Not in foster care (TANF/SCHIP/SSI) | 5,092,574 | 0-5 = 35.5%  6-9 = 22.6%  10-13 = 19.7%  14-18 = 22.1% | Male = 50.9%  Female = 49.1% | White = 41.7%  Black/African American = 29.1%  Hispanic = 24.1%  Other = 5.1% | Not reported |
| Vish (2024); United States | Retrospective cohort study (1 US state; 2017-2020) | Electronic medical records from the healthcare system and referrals from the county health department | Participants: Children in foster care referred to emergency departments and psychiatric hospitals  Criteria: 4-18 years old | Not applicable | 311 | 4.0-7.9 = 106 (34.1%)  8.0-11.9 = 76 (24.4%)  12.0-18.0 = 129 (41.5%) | Male = 161 (51.8%)  Female = 148 (47.6%)  Non-binary = 2 (0.6%) | White = 192 (61.9%)  Black = 108 (34.8%)  Bi-racial = 10 (3.2%) | Developmental delay = 61 (19.6%)  Autism = 7 (2.3%) |
| Zima (1999a); United States | Cross-sectional study (1 US state; 1996-1998) | Structured survey interviews with children in foster homes | Participants: Children in foster care  Criteria: 6-12 years old | Not applicable | 302 | M = 8.5 (SD = 1.8) | Male = 143 (47%)  Female = 159 (53%) | African American = 93 (31%)  Latino = 101 (34%)  Caucasian = 55 (18%)  Biracial = 39 (13%) | ADHD = 37 (15%)  Bipolar = 1 (0%)  Major depression = 13 (5%)  Disorder not specified = 2 (1%) |
| Zima (1999b); United States | Cross-sectional study (1 US state; 1996-1998) | Home interviews with foster parents and children | Participants: Children in foster care  Criteria: 6-12 years old | Not applicable | See Zima (1999a) | See Zima (1999a) | See Zima (1999a) | See Zima (1999a) | See Zima (1999a) |
| Zito (2008); United States | Cross-sectional study (1 US state; 2004) | Medicaid data files | Participants: Children in foster care  Criteria: 0-19 years, receiving psychotropic medication | Not applicable | 472 | 0-4 = 23 (4.9%)  5-9 = 104 (22.0%)  10-14 = 184 (39.0%)  15-19 = 161 (34.1%) | Male = 260 (55.1%)  Female = 212 (44.9%) | White = 181 (38.3%)  Black = 119 (25.2%)  Hispanic = 167 (35.4%)  Other = 5 (1.1%) | ADHD = 38.8%  Bipolar disorder = 17.2%  Depression = 35.5%  Adjustment/anxiety = 33.7%  Oppositional defiant disorder/conduct disorder = 20.6% |
| Zito (2005); United States | Cross-sectional study (1 US state; 2000) | Medicaid pharmacy claims and encounter data | Participants: Medicaid-enrolled youth  Criteria: 2-19 years old | Group 1: Foster care | 13,172 | 2-4 = 10.0%  5-9 = 31.7%  10-14 = 34.2%  15-19 = 24.1% | Male = 51.1%  Female = 48.9% | White = 23.3%  Black = 73.2%  Other = 3.5% | Not reported |
|  |  |  |  | Group 2: Temporary Assistance to Needy Families (TANF) | 96,116 | 2-4 = 9.6%  5-9 = 39.9%  10-14 = 34.3%  15-19 = 16.2% | Male = 47.0%  Female = 52.9% | White = 24.0%  Black = 68.4%  Other = 7.6% | Not reported |
|  |  |  |  | Group 3: State Children’s Health Insurance Program (S-CHIP) | 94,933 | 2-4 = 35.3%  5-9 = 26.9%  10-14 = 20.5%  15-19 = 17.3% | Male = 49.6%  Female = 50.4% | White = 37.8%  Black = 45.9%  Other = 16.3% | Not reported |
|  |  |  |  | Group 4: Supplemental Security Income (SSI) | 13,679 | 2-4 = 9.5%  5-9 = 25.9%  10-14 = 33.6%  15-19 = 31.0% | Male = 64.0%  Female = 36.0% | White = 31.1%  Black = 62.8%  Other = 6.1% | Not reported |
| Zito (2013); United States | Cross-sectional study (1 US state; 1997-2006) | Medicaid administrative claims data | Participants: Medicaid-enrolled youth  Criteria: 2-17 years old | Group 1: Foster care | 14,854 | 2-4 = 65,331 (22.0%)  5-9 = 95,068 (32.0%)  10-14 = 88,240 (29.7%)  15-17 = 48,505 (16.3%) | Male = 150,596 (50.7%)  Female = 146,548 (49.3%) | White = 81,237 (27.3%)  African American = 166,039 (55.9%)  Hispanic = 32,694 (11.0%)  Other = 8,179 (2.8%)  Unknown = 8,995 (3.0% | Schizophrenia or other psychosis = 239 (83.6%)  Pervasive developmental disorder and mental retardation = 199 (38.6%)  Bipolar disorder = 1,418 (72.1%)  Disruptive behaviour = 1,552 (22.1%)  Depressive disorder = 1,103 (21.5%)  Anxiety disorder = 206 (12.4%)  Adjustment disorder = 261 (4.8%)  Communication or learning disorder = 12 (0.9%)  Other = 94 (24.4%) |
|  |  |  |  | Group 2: Temporary Assistance to Needy Families (TANF) | 155,933 |  |  |  |  |
|  |  |  |  | Group 3: State Children’s Health Insurance Program (S-CHIP) | 110,141 |  |  |  |  |
|  |  |  |  | Group 4: Supplemental Security Income (SSI) | 16,216 |  |  |  |  |

Supplemental Table 2. Prevalence of overall psychotropic medication use (n = 44)

| **Study** | **Sample Group** | **Sample Size** | **Any Psychotropic Mediation: n (%)** | **Significant Predictors/Predisposing Factors** |
| --- | --- | --- | --- | --- |
| Bozzi (2022) | Total: Youth in foster care | 4,334 | 1,904 (44%) | - Community adversity |
|  | Group 1: Low community adversity | 1,117 | 540 (48%) |  |
|  | Group 2: Medium community adversity | 1,389 | 738 (53%) |  |
|  | Group 3: High community adversity | 1,828 | 626 (34%) |  |
| Breland-Noble (2004) | Group 1: Therapeutic foster care | 184 | 123 (67%) | - Age (< 13 years) - Ethnicity (white) - Behaviour (externalising) - Behaviour (externalising +\ internalising) |
|  | Group 2: Group homes | 120 | 92 (77%) |  |
| Brenner (2014) | Treatment foster care | 240 | 142 (59.2%) | - Age (6-12 years) - Months in TFC home - Psychopathology (SDQ total) - Service use (case management, social services, and school services) |
| Bush (2021) | Foster children with combined stimulants and antipsychotics | 21 | 21 (100%) | - |
| Chiang (2024) | Group 1: Foster care | 7,369 | 7,369 (100%) | - |
|  | Group 2: Low income | 79,064 | 79,064 (100%) |  |
|  | Group 3: Children’s Health Program (CHP) | 25,425 | 25,425 (100%) |  |
|  | Group 4: Youth with disabilities | 15,114 | 15,114 (100%) |  |
|  |  |  |  |  |
| Cosme (2024) | Children in foster care receiving treatment from a psychiatric institution | 672 | 273 (40.63%) | - Age |
| Davis (2021) | Group 1: Foster care with high level polypharmacy | 417 | 417 (100%) | - |
|  | Group 2: Not in foster care with high level polypharmacy | 1,823 | 1,823 (100%) |  |
| Desjardins (2017) | Children in foster care | 101 | 71 (70.3%) | - Psychopathology symptoms (CBCL) (Social disorders, Aggressive behaviours, Attention deficit problems, Anxiety-depression, Thoughts disorders, Sexual disorders behaviours, Post-traumatic disorders, Internalised disorders, Externalised disorders) - Overall functioning (CAFAS) (Functioning within family, Behaviour towards others, Moods, Thought, Total) - *Post-traumatic stress symptoms (CBCL)* |
| dosReis (2014) | Children in foster care | 1,491 | 178 (11.9%) | - |
| dosReis (2011) | Group 1: Foster care | 2,310 | 2,310 (100%) | - |
|  | Group 2: Supplemental Security Income (SSI) | 8,787 | 8,787 (100%) |  |
|  | Group 3: Temporary Assistance Families (TANF) | 3,631 | 3,631 (100%) |  |
| dosReis (2001) | Group 1: Foster care | 310 | 94 (20.6%) | - |
|  | Group 2: Supplemental Security Income (SSI) | 775 | 137 (17.7%) |  |
|  | Group 3: Other Aid | 14,442 | 238 (1.7%) |  |
| Ferguson (2006) | Children in foster care | 473 | 202 (43%) | - Age - Gender (male) - Ethnicity (European American) |
| Fontanella (2015) | Youth in foster care | 952 | 742 (77.94%) | - |
| Fontanella (2014) | Group 1: Foster care | 4,906 | 4,906 (100%) | - |
|  | Group 2: Disability | 8,592 | 8,592 (100%) |  |
|  | Group 3: Low income | 36,813 | 36,813 (100%) |  |
| Gardner (2016) | Group 1: Foster care | 743 | 15 y/old = 47.5%  16 y/old = 48.3%  17 y/old = 42.8%  18 y/old = 29.1% | - Age |
|  | Group 2: Non-foster Medicaid | 2,636 | 15 y/old = 15.4%  16 y/old = 17.3%  17 y/old = 18.6%  18 y/old = 16.8% |  |
|  | Group 3: Private coverage | 1,947 | 15 y/old = 6.7%  16 y/old = 8.9%  17 y/old = 10.5%  18 y/old = 9.8% |  |
| Glesener (2018) | Total: Children in foster care | 626 | 162 (25.95% | - Age (15-17 years) - Gender (male) - Race (European American) - Time in foster care |
|  | Group 1: European American | 332 | 97 (29.2%) |  |
|  | Group 2: American Indian | 223 | 52 (23.3%) |  |
|  | Group 3: African American | 71 | 13 (18.3%) |  |
| Green (2005) | Children in foster care | 722 | 159 (23%) | - |
| Keast (2019) | Group 1: Foster care | 9,325 | 7,264 (77.9%) | - |
|  | Group 2: Not in foster care | 639,868 | 399,206 (62.4%) |  |
| Leathers (2021) | Children in foster care | 144 | 75 (52.1%) | - Gender (male) |
| Linares (2013) | Children in foster care | 252 | 75 (31%) | - |
| McLeigh (2023) | Children in foster care | 3,067 | 851 (27.8%) | - Number of maltreatment exposure types (at least 1) - Maltreatment exposure (yes) - Sexual abuse (no) - Physical abuse (no) - Neglect (yes) - Physical health status - Time in care |
| McMillen (2004) | Youth in foster care | 406 | 149 (37%) | - Youth of colour - Age of entrance to foster care system - Disorder in the past 12 months - Congregate care placement |
| Narendorf (2011) | Youth in treatment foster care | 8 | 8 (100%) | - |
| Nunes (2022) | Time 1: Youth in foster care in 2011 | 78,231 | 10,435 (13.3%) | - Age group (16-17 years) - Gender (male) - Race/ethnicity (black/white) |
|  | Time 2: Youth in foster care in 2020 | 68,386 | 7,172 (12.2%) |  |
| Oerbeck (2021) | Children and adolescents in residential care institutions (0-20 years) | 1,856 | 423 (23%) | - |
| Palmer (2023) | Early adolescents in foster care | 2,998 | 1,259 (42.0%) | - Age at current removal - Gender (male) - Race/ethnicity (white) - Residential care placement - History of neglect, physical, and sexual abuse - Prior removals |
| Park (2019) | Transition age foster youth | 611 | 117 (27.2%) | - Any mental health or substance use disorder - Congregate care or independent living placement |
| Raghavan (2010) | Children in the child welfare system | 3,017 | 501 (14.1%) | - Age - Gender (male) - Race/ethnicity (white) - Current placement out-of-home - Insurance status (private, federal, or uninsured) - Externalising or internalising behaviour |
| Raghavan (2008) | Transition age foster youth | 403 | 146 (36.2%) | - |
| Rose (2023) | Youth in foster care with disruptive behaviour disorders following ADHD diagnosis | 10,005 | 1,571 (15.7%) | - |
| Rubin (2012) ^a^ | Time 1: Youth in foster care in 2002 | 91.4 | 3-5: 6.7 (7.4%)  6-11: 61.5 (26.6%)  12-18: 99.4 (31.9%) | - |
|  | Time 2: Youth in foster care in 2007 | 108.4 | 3-5: 7.1 (6.5%)  6-11: 66.3 (27.0%)  12-18: 123.1 (33.0%) |  |
| Rubin (2009) | Group 1: Foster care | N/R | 71.6% | - |
|  | Group 2: Supplemental Security Income (SSI) | N/R | 56.3% |  |
| Scorzzaro (2015) | Children in foster care | 128 | 32 (42%) | - |
| Spence (2019) | Children in foster care | 753 | 753 (100%) | - |
| Solerdelcoll (2022) | Group 1: Youth in foster care in an inpatient psychiatric ward | 89 | 88 (98.9%) | - |
|  | Group 2: Non-foster youth living at home | 247 | 220 (89.1%) |  |
| Soto-Ramirez (2024) | Children in foster care | 1,002 | 950 (94.8%) | - |
| Tai (2016) | Youth in foster care who initiated antipsychotic treatment | 390 | 390 (100%) | - |
| Tan (2023) | Group 1: Foster care | 2,758 | 1,214 (44.02%) | - |
|  | Group 2: Comparison youth not in foster care | 2,787 | 781 (28.02%) |  |
| Vish (2024) | Children in foster care referred to emergency departments and psychiatric hospitals | 311 | 88 (28.3%) | - Mental health ED visits - Psychiatric hospitalisation |
| Zima (1999a) | Children in foster care | 302 | 49 (16%) | - Ethnicity (Caucasian or biracial) - Group home placement |
| Zima (1999b) | See Zima (1999a) | See Zima (1999a) | Zima (1999b) | - |
| Zito (2008) | Children in foster care receiving psychotropic medication | 472 | 472 (100%) | - |
| Zito (2005) | Group 1: Foster care | 13,172 | 25.8% | - Race/ethnicity (white) |
|  | Group 2: Temporary Assistance to Needy Families (TANF) | 96,116 | 7.4% |  |
|  | Group 3: State Children’s Health Insurance Program (S-CHIP) | 94,933 | 6.0% |  |
|  | Group 4: Supplemental Security Income (SSI) | 13,679 | 34.7% |  |

Supplemental Table 3. Prevalence of psychotropic medication use across subclasses (n = 44)

| **Study** | **Sample Group** | **Sample Size** | **Psychotropic Medication Subclasses: n (%)** | | | | | | **Significant Predictors/Predisposing Factors** |
| --- | --- | --- | --- | --- | --- | --- | --- | --- | --- |
|  |  |  | **Stimulants and/or other ADHD Medication** | **Antipsychotics** | **Antidepressants** | **Anxiolytics** | **Mood Stabilisers** | **Other** |  |
| Allaire (2016) | Group 1: Obesity diagnosis | 6,517 | - | 19.5% | - | - | - | - | - |
|  | Group 2: No obesity diagnosis | 1,255,289 | - | 19.5% | - | - | - | - |  |
| Bozzi (2022) | Total: Youth in foster care | 4,334 | 1,355 (31%) | 874 (20%) | 891 (21%) | 144 (3%) | 496 (11%) | - | - |
|  | Group 1: Low community adversity | 1,117 | 372 (33%) | 268 (6%) | 266 (24%) | 52 (5%) | 140 (13%) | - |  |
|  | Group 2: Medium community adversity | 1,389 | 520 (37%) | 360 (26%) | 367 (26%) | 53 (4%) | 214 (15%) | - |  |
|  | Group 3: High community adversity | 1,828 | 463 (25%) | 246 (13%) | 258 (14%) | 39 (2%) | 142 (8%) | - |  |
| Brenner (2014) | Treatment foster care (n = 240) | 240 | - | 68 (26%) | 54 (22%) | 0 (0%) | 29 (12%) | - | ADHD medication:   - Age (6-12 years) |
| Burcu (2014) | Group 1: Foster care | N/R | - | 2,224 | - | - | - | - | Antipsychotics   - *Age groups (2-12 years)* - *Gender (male)* - *Ethnicity (white)* - *Diagnosis (schizophrenia / other psychoses)* |
|  | Group 2: Children’s Health Insurance Program (CHIP) | N/R | - | 1,184 | - | - | - | - |  |
|  | Group 3: Temporary Assistance Families (TANF) | N/R | - | 2,839 | - | - | - | - |  |
|  | Group 4: Supplemental Security Income (SSI) | N/R | - | 2,449 | - | - | - | - |  |
| Bush (2021) | Foster children with combined stimulants and antipsychotics | 21 | 21 (100%) | 21 (100%) | - | - | - | - | - |
| Cosme (2024) | Children in foster care receiving treatment from a psychiatric institution | 672 | Stimulants: 91 (13.5%)  Non-stimulants: 179 (26.6%) | 126 (18.8%) | 117 (17.4%) | 26 (4.2%) | 28 (4.2%) | - | - |
| Crystal (2016) | Group 1: Medicaid-insured children in foster care | N/R | - | 2005: 8.73%  2008: 9.26%  2010: 8.92% | - | - | - | - | - |
|  | Group 2: Medicaid-insured children not in foster care | N/R | - | 2008: 1.86%  2010: 1.73% | - | - | - | - |  |
| Desjardins (2017) | Children in foster care | 101 | 65 (92.9%) | 36 (51.4%) | 8 (11.4%) | - | 1 (1.43%) | Alpha-adrenergic agonists = 13 (18.6%)  Anticonvulsants = 1 (1.4%) | - |
| dosReis (2014) | Children in foster care | 1,491 | 70 (5%) | 28 (2%) | 17 (1%) | - | 12 (<1%) | Alpha agonists: 10 (<1%) | Antipsychotics:   - Age - Mood disorder - Antidepressant or ADHD prescription   ADHD medication:   - Age - Disruptive behaviour disorder   Mood stabilisers:   - Disruptive behaviour disorder   Antidepressants:   - Age - Internalising disorder |
| dosReis (2011) | Group 1: Foster care | 2,310 | 1,142 (49.4%) | 2,256 (97.7%) | 1,377 (59.6%) | - | 986 (42.7%) | - | - |
|  | Group 2: Supplemental Security Income (SSI) | 8,787 | 5,085 (57.9%) | 8,650 (98.4%) | 4,719 (53.7%) | - | 3,577 (40.7%) | - |  |
|  | Group 3: Temporary Assistance Families (TANF) | 3,631 | 1,992 (54.9%) | 3,604 (99.3%) | 1,946 (53.6%) | - | 873 (24.0%) | - |  |
| dosReis (2001) | Group 1: Foster care | 310 | 57 (18%) | 18 (6%) | 47 (15%) | 6 (2%) | - | Anticonvulsants: 21 (7%)  Antiparkinsonian: 4 (1%  Clonidine: 15 (5%)  Lithium: 7 (2%) | Stimulants, antidepressants, and anticonvulsants:   - Ethnicity (white) |
|  | Group 2: Supplemental Security Income (SSI) | 775 | 51 (7%) | 37 (5%) | 56 (7%) | 22 (3%) | - | Anticonvulsants: 61 (8%)  Antiparkinsonian: 13 (2%)  Clonidine: 18 (2%)  Lithium: 14 (2%) |  |
|  | Group 3: Other Aid | 14,442 | 152 (1.0%) | 24 (0.2%) | 76 (0.5%) | 9 (0.06%) | - | Anticonvulsants: 52 (0.4%)  Antiparkinsonian: 6 (0.04%)  Clonidine: 18 (0.1%)  Lithium: 12 (0.08%) |  |
| Ferguson (2006) | Children in foster care | 473 | 114 (56%) | 35 (17%) | 126 (62%) | 24 (12%) | 28 (14%) | Antihypertensives: 24 (12%)  Lithium: 6 (3%) | No significant predictors |
| Fontanella (2014) | Group 1: Foster care | 4,906 | 3,687 (75.2%) | 1,949 (39.7%) | 1,177 (24.0%) | 84 (1.7%) | 967 (19.7%) | Alpha-agonists: 910 (18.6%) | - |
|  | Group 2: Disability | 8,592 | 4,921 (57.3%) | 3,102 (36.1%) | 1,530 (17.8%) | 723 (8.4%) | 2,801 (32.6%) | Alpha-agonists: 2,006 (23.4%) |  |
|  | Group 3: Low income | 36,813 | 28,231 (76.7%) | 7,102 (19.3%) | 6,081 (16.5%) | 666 (1.8%) | 4,601 (12.5%) | Alpha-agonists: 5,957 (16.2%) |  |
| Glesener (2018) | Total: Children in foster care | 626 | 89 (14.2%) | 32 (5.1%) | 87 (13.9%) | - | - | Alpha-agonists: 45 (7.2%)  Other: 30 (4.8%) | Antidepressants:   - Age (15-17 years) - Time in foster care   ADHD medication:   - Age (15-17 years) - Gender (male) - Race (European American) - Time in foster care   Alpha-agonists:   - Gender (male)   Antipsychotics:   - Age (15-17 years) - Gender (male) |
|  | Group 1: European American | 332 | 60 (18.1%) | 22 (6.6%) | 49 (14.8%) | - | - | Alpha-agonists: 28 (8.4%)  Other: 20 (6.0%) |  |
|  | Group 2: American Indian | 223 | 24 (10.8%) | 8 (3.6%) | 30 (13.5%) | - | - | Alpha-agonists: 15 (6.7%)  Other: 9 (4.0%) |  |
|  | Group 3: African American | 71 | 5 (7.0%) | 2 (2.8%) | 8 (11.3%) | - | - | Alpha-agonists: 2 (2.8%)  Other: 1 (1.4%) |  |
| Green (2005) | Children in foster care | 722 | 53 (7.3%) | 41 (5.7%) | 21 (2.9%) | - | - | Antihypertensives: 21 (2.9%)  Anticonvulsants: 31 (4.3%)  Antihistamines: 13 (1.8%)  SSRIs: 29 (4.0%) | - |
| Keast (2019) | Group 1: Foster care | 9,325 | 1,579 (16.9%) | 808 (8.7%) | 1,113 (11.9%) | 433 (4.64%) | 451 (4.8%) | - | - |
|  | Group 2: Not in foster care | 639,868 | 42,317 (6.6%) | 10,724 (1.7%) | 26,998 (4.2%) | 16,502 (2.6%) | 6,829 (1.1%) | - |  |
| Leathers (2021) | Children in foster care | 144 | 62 (43.1%) | 29 (20.1%) | 18 (12.5%) | - | 6 (4.2%) | - | - |
| Leckman-Westin (2018) | Group 1: Foster care | 31,375 | - | Antipsychotics: 31,375 (100%)  Higher than recommended dose: 5,130 (10.4%) | - | - | - | - | - |
|  | Group 2: Supplemental Security Income (SSI) | 47,694 | - | Antipsychotics: 47,694 (100%)  Higher than recommended dose: 5,130 (11.3%) | - | - | - | - |  |
|  | Group 3: Other Aid | 6,289 | - | Antipsychotics: 6,289 (100%)  Higher than recommended dose: 238 (4.2%) | - | - | - | - |  |
|  | Group 4: Income Aid | 58,842 | - | Antipsychotics: 58,842 (100%)  Higher than recommended dose: 2,632 (4.9%) | - | - | - | - |  |
| Linares (2013) | Children in foster care | 252 | Stimulants: 52 (69%)  Non-stimulants: 19 (25%) | Atypical: 49 (65%)  Typical: 1 (0%) | Antidepressants / anxiolytics: 5 (7%) | Antidepressants / anxiolytics: 5 (7%) | 10 (13%) | Other: 3 (4%) | Stimulants:   - Gender (male) - Ethnicity (African American): - Diagnoses (ADHD + ODD or CD, ADHD +/-. Oppositional defiant, Conduct, Separation anxiety) - Behaviour (ECBI-PR Aggression+)   Antipsychotics:   - Gender (male) - Ethnicity (African American) - Diagnoses (ADHD + ODD or CD, ADHD +/-, Oppositional defiant, Conduct, Major depression) - Maltreatment history (Sexual abuse) |
| McLeigh (2023) | Children in foster care | 3,067 | Stimulants: 315 (10.3%)  Non-stimulants: 386 (12.6%) |  | Antidepressants / anxiolytics: 116 (3.8%) | Antidepressants / anxiolytics: 116 (3.8%) | 395 (12.9%) | Hypnotics: 59 (1.9%)  Benzodiazepines: 15 (0.5%)  Other: 116 (3.8%) | - |
| McMillen (2004) | Youth in foster care | 406 | 34 (8%) | 77 (19%) | 106 (26%) | 28 (7%) | - | Antimanic properties: 71 (18%) | - |
| Narendorf (2011) | Youth in treatment foster care | 8 | 6 (75%) | 8 (100%) | 8 (100%) | 3 (37.5%) | 6 (75%) | Anticonvulsants: 4 (50%)  Antihypertensives: 5 (62.5%)  Opioid antagonists: 1 (12.5%)  Sedatives/hypnotics: 1 (12.5%) | - |
| Nunes (2022) | Time 1: Youth in foster care in 2011 | 78,231 | - | 5,570 (7.1%) | - | - | - | - | - |
|  | Time 2: Youth in foster care in 2020 | 68,386 | - | 2,068 (3%) | - | - | - | - |  |
| Oerbeck (2020) ^a^ | Children and adolescents in residential care | 2,171 | 19% | 25% | 23% | 9% | - | Sleep medication: 17% | Sleep medication:   - Age group (increased with age) - Gender (female) |
| Oerbeck (2021) | Children and adolescents in residential care institutions (0-20 years) | 1,856 | 178 (9.6%) | 200 (10.8%) | 165 (8.9%) | - | - | - | Not applicable |
| Monson (2023) | Youth in foster care | 8,523 | - | 757 (8.9%) | - | - | - | - | Antipsychotics:   - Diagnostic indication (mood, gastrointestinal, trauma-associated, behavioural disorders) - Concurrent medication classes (alpha modulators, antiepileptic / mood stabilisers, laxatives / bowel regimen) - Reasons for placement (parent condition, ungovernable) |
| Palmer (2023) | Early adolescents in foster care | 2,998 | 26.7% | 18.7% | - | - | - | - | - |
| Raghavan (2008) | Transition age foster youth | 403 | - | 72 (18%) | 34 (63%) | - | 55 (14%) | - | - |
| Robst (2009) | Group 1: Therapeutic foster care | 860 | - | 40% | - | - | - | - | - |
|  | Group 2: Statewide Inpatient Psychiatric Program (SIPP) | 1,080 | - | 60% | - | - | - | - |  |
|  | Group 3: Therapeutic Group Care (TGC) | 489 | - | 50% | - | - | - | - |  |
| Rose (2022) | Youth in foster care with disruptive behaviour disorders | 10,397 | Stimulants: 3,401 (33%)  Non-stimulants: 2,179 (21%) | 2,928 (28%) | 1,350 (13%) | 210 (2%) | 523 (5%) | - | Antipsychotics:   - Services received prior to prescribing (crisis services, multisystemic therapy, intensive in-home services, child and adolescent day treatment, case management, psychiatry, psychotherapy, community / behavioural health, comprehensive outpatient rehabilitation) |
| Rose (2023) | Youth in foster care with disruptive behaviour disorders following ADHD diagnosis | 10,005 | Stimulants: 5,115 (51%)  Non-stimulants: 894 (16.9%) | 1,571 (15.7%) | 577 (5.8%) | 49 (0.5%) | 176 (1.8%) | Sedatives: 8 (0.1%) | Antipsychotics:   - Baseline prescriptions (mood stabiliser, antidepressants) - Baseline diagnosis (disruptive behaviour, anxiety, suicide attempt or self-injury, adjustment disorder) |
| Rubin (2012) ^b^ | Time 1: Youth in foster care in 2002 | 91.4 | 3-5: 4.2 (4.6%)  6-11: 48.3 (20.9%)  12-18: 48.0 (15.4%) | 3-5: 1.6 (1.7%)  6-11: 18.2 (7.9%)  12-18: 36.3 (11.7%) | 3-5: 1.5 (1.7%)  6-11: 23.5 (10.2%)  12-18: 61.9 (19.9%) | - | 3-5: 1.3 (1.4%)  6-11: 10.6 (4.6%)  12-18: 27.5 (8.8%) | Alpha agonist:  3-5: 1.6 (1.8%)  6-11: 13.8 (6.0%)  12-18: 12.5 (4.0%) | - |
|  | Time 2: Youth in foster care in 2007 | 108.4 | 3-5: 4.6 (4.2%)  6-11: 55.2 (22.5%)  12-18: 72.8 (19.5%) | 3-5: 2.4 (2.2%)  6-11: 24.8 (10.1%)  12-18: 58.3 (15.6%) | 3-5: 0.9 (0.8%)  6-11: 14.0 (5.7%)  12-18: 53.7 (14.4%) | - | 3-5: 1.1 (1.0%)  6-11: 9.8 (4.0%)  12-18: 32.1 (8.6%) | Alpha agonist:  3-5: 2.1 (2.0%)  6-11: 17.4 (7.1%)  12-18: 17.8 (4.8%) |  |
| Rubin (2009) | Group 1: Foster care | N/R | 37.1% | - | 36.2% | 12.2% | - | Neuroleptics: 45.6%  Anticonvulsants: 30.5%  Hypnotics: 3.3% | - |
|  | Group 2: Supplemental Security Income (SSI) | N/R | 22.3% | - | 23.8% | 11.7% | - | Neuroleptics: 30.7%  Anticonvulsants: 20.9%  Hypnotics: 3.2% |  |
| Spence (2019) | Children in foster care | 753 | - | 753 (100%) | - | - | - | - | - |
| Solerdelcoll (2022) | Group 1: Youth in foster care in an inpatient psychiatric ward | 89 | 10 (11.2%) | Any: 85 (95.5%)  Second generation LAIs: 22 (24.7%) | 26 (29.2%) | - | 20 (22.5%) | - | - |
|  | Group 2: Non-foster youth living at home | 247 | 16 (6.5%) | Any antipsychotics: 186 (75.4%)  Second generation LAIs: 14 (5.7%) | 120 (48.6%) | - | 37 (15.0%) | - |  |
| Tai (2016) | Youth in foster care who initiated antipsychotic treatment | 390 | - | 65 (16.7%) | - | - | - | - | - |
| Tan (2023) | Group 1: Foster care | 2,758 | - | 682 (6.32%) | - | - | - | - | Antipsychotics:   - No. mental health diagnoses - Service use (inpatient care) - No. placements before first antipsychotic prescribed |
|  | Group 2: Comparison youth not in foster care | 2,787 | - | 176 (6.32%) | - | - | - | - |  |
| Vanderwerker (2014) | Group 1: Foster care | 301,894 | - | 7.4% | - | - | - | - | - |
|  | Group 2: Not in foster care (TANF/SCHIP/SSI) | 5,092,574 | - | 1.4% | - | - | - | - |  |
| Zima (1999a) | Children in foster care | 302 | 24 (62%) | 3 (8%) | 12 (31%) | 3 (8%) | 12 (31%) | Alpha agonist: 10 (26%)  Antihistamine: 1 (3%)  Anticholinergic: 2 (5%) | - |
| Zima (1999b) | See Zima (1999a) | Zima (1999b) | See Zima (1999a) | Zima (1999b) | See Zima (1999a) | Zima (1999b) | See Zima (1999a) | Zima (1999b) | Stimulants:   - Diagnoses (ADHD, major depression)   Alpha agonists:   - Diagnoses (ADHD)   Antidepressants:  No significant predictors  Mood stabilisers:   - Diagnoses (ADHD)   Anxiolytics:   - Diagnoses (ADHD)   Antipsychotics:   - Diagnoses (ADHD, psychotic disorder) |
| Zito (2008) | Children in foster care receiving psychotropic medication | 472 | 264 (55.9%) | 251 (53.2%) | 268 (56.8%) | 22 (4.7%) | - | Alpha agonists: 84 (17.8%)  ATC-MSs: 132 (28.0%)  Lithium: 11 (2.3%)  Miscellaneous: 64 (13.6%) | - |
| Zito (2005) | Group 1: Foster care | 13,172 | 17.2% | - | 11.3% | - | - | Neuroleptics: 7.7% | - |
|  | Group 2: Temporary Assistance to Needy Families (TANF) | 96,116 | 5.4% | - | 2.4% | - | - | Neuroleptics: 0.9% |  |
|  | Group 3: State Children’s Health Insurance Program (S-CHIP) | 94,933 | 7.7% | - | 2.0% | - | - | Neuroleptics: 2.0% |  |
|  | Group 4: Supplemental Security Income (SSI) | 13,679 | 17.7% | - | 12.8% | - | - | Neuroleptics: 11.9% |  |
| Zito (2013) | Group 1: Foster care | 14,854 | - | 2,431 (16.4%) | - | - | - | - | - |
|  | Group 2: Temporary Assistance to Needy Families (TANF) | 155,933 | - | 3,072 (2.0%) | - | - | - | - |  |
|  | Group 3: State Children’s Health Insurance Program (S-CHIP) | 110,141 | - | 1,303 (1.2%) | - | - | - | - |  |
|  | Group 4: Supplemental Security Income (SSI) | 16,216 | - | 2,750 (17.0%) | - | - | - | - |  |

Supplemental Table 4. Prevalence of multiple medication use across studies (n = 29)

| **Study** | **Sample Group** | **Sample Size** | **Polypharmacy Rates** | **Significant Predictors/Predisposing Factors** |
| --- | --- | --- | --- | --- |
| Allaire (2016) | Group 1: Obesity diagnosis | 6,517 | None = 79.1%  One = 19.5%  Two or more = 1.4% | - |
|  | Group 2: No obesity diagnosis | 1,255,289 | None = 90.5%  One = 9.2%  Two or more = 0.3% |  |
| Breland-Noble (2004) | Group 1: Therapeutic foster care | 184 | Four or more = 11 (6%) | - Age (< 13 years) - Behaviour (externalising + internalising) |
|  | Group 2: Group homes | 120 | Four or more = 18 (15%) |  |
| Brenner (2014) | Treatment foster care | 240 | One = 56 (23.3%)  Two = 51 (21.2%)  Three = 22 (9.2%)  Four or more = 13 (5.4%)  Questionable = 31 (12.9%) | - Psychopathology (SDQ total) - School services - Outpatient treatment |
| Chiang (2024) | Group 1: Foster care | 7,369 | 2015: 414/3824 (10.8%)  2020: 387/3420 (11.3%) | - |
|  | Group 2: Low income | 79,064 | 2015: 648/30,222 (2.1%)  2020: 883/31,172 (2.8%) |  |
|  | Group 3: Children’s Health Program (CHP) | 25,425 | 2015: 225/10,354 (2.2%)  2020: 222 (7,974 (2.8%) |  |
|  | Group 4: Youth with disabilities | 15,114 | 2015: Not reported  2020: Not reported |  |
| Cosme (2024) | Children in foster care receiving treatment from a psychiatric institution | 672 | 30-day concurrent use = 102 (37%)  60-day concurrent use = 76 (28%)  Two = 31 (41%)  Three = 24 (32%)  Four = 15 (20%)  Five = 5 (7%)  Six = 1 (1%) | - |
| Crystal (2016) | Group 1: Medicaid-insured children in foster care | N/R | Two or more antipsychotics:  2009: 3.98%  2010: 3.73%  2011: 2.76% | - |
|  | Group 2: Medicaid-insured children not in foster care | N/R | Two or more antipsychotics:  2009: 3.70%  2010: 3.36%  2011: 3.08% |  |
| Davis (2021) | Group 1: Foster care with high level polypharmacy | 417 | High-level polypharmacy = 417 (100%)  Polypharmacy (2+) duration (days): Mdn = 325.0, IQR = 278.0 – 345.0  Polypharmacy (4+) duration (days): Mdn = 144.0, IQR = 67.0 – 244.0 | - Diagnostic category (other neurodevelopmental, disruptive behaviour disorder, impulse control) |
|  | Group 2: Not in foster care with high level polypharmacy | 1,823 | High-level polypharmacy = 1,823 (100%)  Polypharmacy (2+) duration (days): Mdn = 330.0, IQR = 289.0 – 346.0  Polypharmacy (4+) duration (days): Mdn = 156.0, IQR = 78.0 – 255.0) |  |
| Desjardins (2017) | Children in foster care | 101 | One = 21 (30%)  Two = 30 (42%)  Three = 11 (16%)  Four or more = 9 (13%) | - |
| dosReis (2014) | Children in foster care | 1,491 | Three or more = 11 (<1%) | - |
| dosReis (2011) | Group 1: Foster care | 2,310 | Single use = 1,819 (79%)  Multiple use = 491 (21%) | - |
|  | Group 2: Supplemental Security Income (SSI) | 8,787 | Single use = 7,062 (80%)  Multiple use = 1,725 (20%) |  |
|  | Group 3: Temporary Assistance Families (TANF) | 3,631 | Single use = 3,152 (87%)  Multiple use = 479 (13%) |  |
| Ferguson (2006) | Children in foster care | 473 | Two or more = 108 (53.46%)  Four or more = 18 (8.91%) | - |
| Fontanella (2015) | Youth in foster care | 952 | Zero = 210 (22.1%)  One = 157 (16.5%)  Two or more = 585 (61.5%) | - |
| Fontanella (2014) | Group 1: Foster care | 4,906 | One = 2,261 (46.1%)  Two = 1,308 (26.7%)  Three = 849 (17.3%)  Four = 363 (7.4%)  Five or more = 125 (2.5%) | - |
|  | Group 2: Disability | 8,592 | One = 3,987 (46.4%)  Two = 2,464 (28.7%)  Three = 1,364 (15.9%)  Four = 562 (6.5%)  Five or more = 215 (2.5%) |  |
|  | Group 3: Low income | 36,813 | One = 23,864 (64.8%)  Two = 8,732 (23.7%)  Three = 3,052 (8.3%)  Four = 904 (2.5%)  Five or more = 261 (0.7%) |  |
| Gardner (2016) | Group 1: Foster care | 743 | 15 y/old: Mdn = 5  16 y/old: Mdn = 5  17 y/old: Mdn = 5  18 y/old: Mdn = 4 | - |
|  | Group 2: Non-foster Medicaid | 2,636 | 15 y/old: Mdn = 3  16 y/old: Mdn = 4  17 y/old: Mdn = 4  18 y/old: Mdn = 3 |  |
|  | Group 3: Private coverage | 1,947 | 15 y/old: Mdn = 1  16 y/old: Mdn = 2  17 y/old: Mdn = 2  18 y/old: Mdn = 2 |  |
| Green (2005) | Children in foster care | 722 | One = 43%  Two or more = 57% | - |
| Keast (2019) | Group 1: Foster care | 9,325 | Two = 418 (4.5%)  Three = 310 (3.3%)  Four = 121 (1.3%)  Five or more = 10 (0.1%) | - Age - Gender (male) - Ethnicity (white) - Rural location - Diagnostic comorbidity - Presence of overweight/obesity - Presence of hyperlipidaemia |
|  | Group 2: Not in foster care | 639,868 | Two = 8,010 (1.3%)  Three = 3,366 (0.5%)  Four = 897 (0.1%)  Five or more = 121 (0.0%) |  |
| Leathers (2021) | Children in foster care | 144 | Two or more = 42 (29.2%) | - |
| Leckman-Westin (2018) | Group 1: Foster care | 31,375 | Multiple concurrent antipsychotics = 1,974 (7.9%) | - |
|  | Group 2: Supplemental Security Income (SSI) | 47,694 | Multiple concurrent antipsychotics = 3,221 (8.9%) |  |
|  | Group 3: Other Aid | 6,289 | Multiple concurrent antipsychotics = 154 (4.4%) |  |
|  | Group 4: Income Aid | 58,842 | Multiple concurrent antipsychotics = 1,311 (3.5%) |  |
| Linares (2013) | Children in foster care | 252 | Number of medications:  One = 35 (13.9%)  Two = 26 (10.3%)  Three or more = 14 (0.6%)  Combinations:  Stimulants + antipsychotics = 30 (11.9%) | - |
| McLeigh (2023) | Children in foster care | 3,067 | Zero = 2,216 (72.3%)  One = 463 (15.1%)  Two = 252 (8.2%)  Three = 115 (3.8%)  Four = 15 (0.5%)  Five = 6 (0.2%) | - |
| Narendorf (2011) | Youth in treatment foster care | 8 | One = 1 (12.5%)  Three = 3 (37.5%)  Four = 2 (25%)  Five = 1 (12.5%)  Six = 1 (12.5%) | - |
| Oerbeck (2021) | Children and adolescents in residential care institutions (0-20 years) | 1,856 | One = 312 (74%)  Two = 102 (24%)  Three = 9 (2%) | - |
| Palmer (2023) | Early adolescents in foster care | 2,998 | Polypharmacy = 23.4% | - |
| Raghavan (2008) | Transition age foster youth | 403 | Zero = 257 (63.8%)  One = 54 (13.4%)  Two = 46 (11.4%)  Three or more = 46 (11.4%)  Three = 21 (5%)  Four = 18 (4%)  Five = 7 (2%) | Two concurrent psychotropic medications   - Race/ethnicity (white) - Diagnosis (major depressive disorder and PTSD)   Three or more concurrent psychotropic medications   - Race/ethnicity (white) - Current residential status (living with birthparents, other relatives, or independently) - Maltreatment history (sexual and physical abuse) - Service use (specialty service only) - Diagnosis (manic episode, major depressive disorder, and disruptive behaviour disorder, PTSD) |
| Rubin (2012) ^a^ | Time 1: Youth in foster care in 2002 (n = 91.4) |  | 3-5 vs 6-11 vs 12-18 years:  Polypharmacy (3 or more for at least 30 days): 0.6 (0.7%), 11.8 (5.1%), 20.5 (6.6%) | - |
|  | Time 2: Youth in foster care in 2007 (n = 108.4) |  | 3-5 vs 6-11 vs 12-18 years:  Polypharmacy (3 or more for at least 30 days): 0.7 (0.6%), 11.9 (4.9%), 26.1 (7.0%) |  |
| Rubin (2009) | Group 1: Foster care | N/R | Zero = 28.4%  One = 20.1%  Two = 22.8%  Three or more = 28.7%  Concurrent use of three or more = 20.8% | - |
|  | Group 2: Supplemental Security Income (SSI) | N/R | Zero = 43.7%  One = 22.2%  Two = 18.1%  Three or more = 15.9%  Concurrent use of three or more = 10.1% |  |
| Spence (2019) | Children in foster care | 753 | Single class = 304 (40.4%)  Two or more classes = 170 (22.6%) | - Number of placement changes - Number of diagnoses - Diagnoses (bipolar/mood, depression, ADHD, conduct, oppositional defiant, anxiety, PTSD) |
| Zima (1999a) | Children in foster care | 302 | No. medications taken: M = 2.1 (SD = 1.4)  One = 20 (51%)  Two = 8 (21%) | - |
| Zito (2008) | Children in foster care receiving psychotropic medication | 472 | Average number of medications per child = 2.55  One = 130 (27.5%)  Two = 147 (31.1%)  Three = 120 (25.4%)  Four = 65 (13.8%)  Five or more = 10 (2.1%) | - |

**Forest plots** **illustrating prevalence estimates across five psychotropic subclasses**


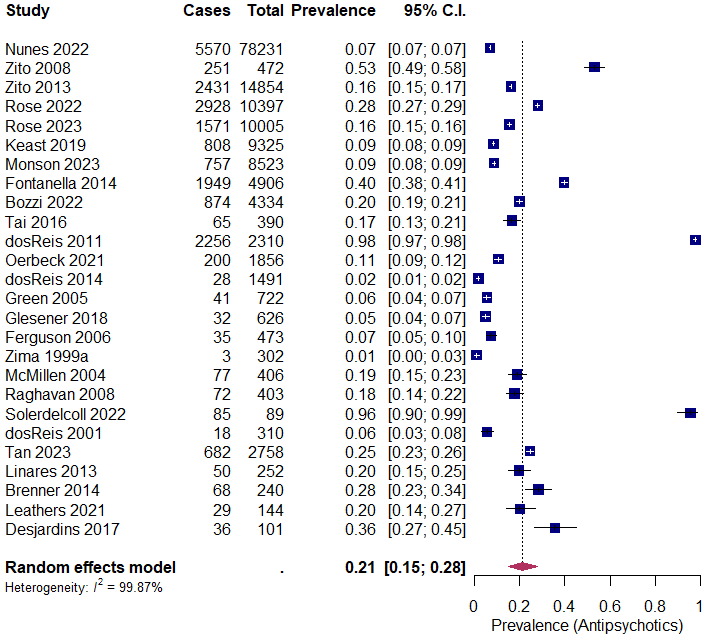


*Figure S1. Forest plot illustrating the pooled prevalence of antipsychotic use*


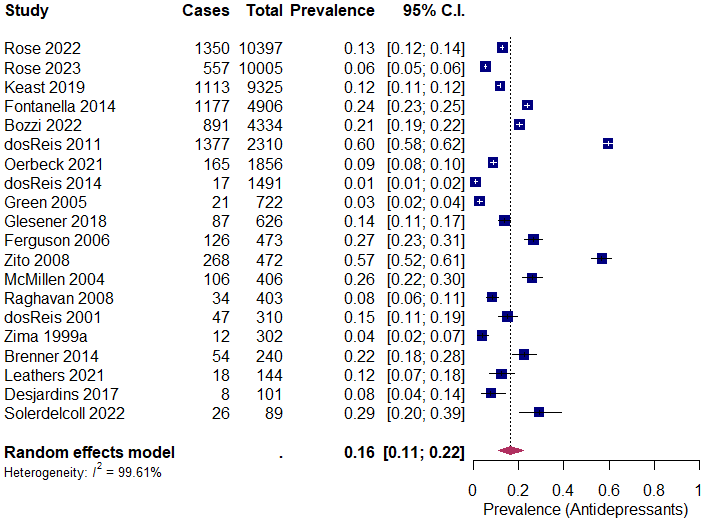


*Figure S2. Forest plot illustrating the pooled prevalence of antidepressant use*


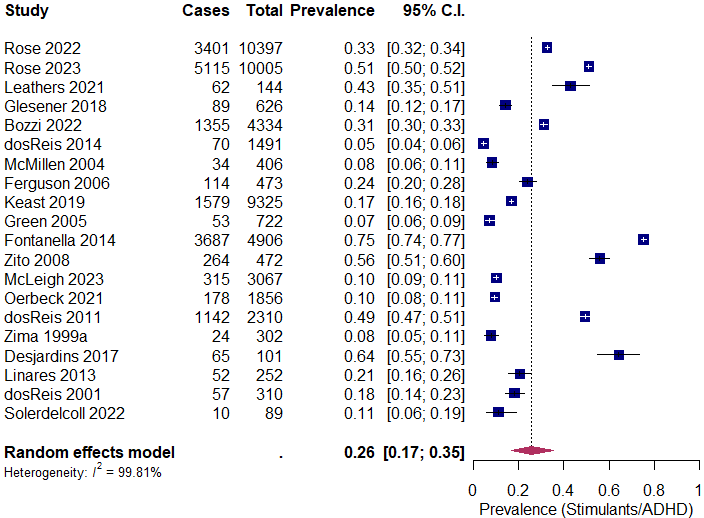


*Figure S3. Forest plot illustrating the pooled prevalence of stimulant and other ADHD medication use*


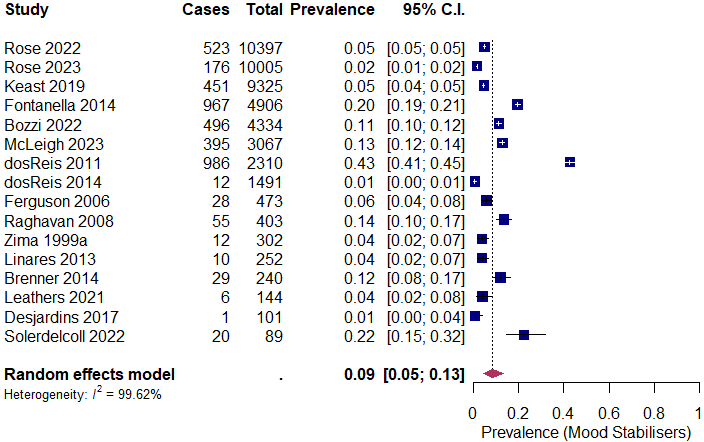


*Figure S4. Forest plot illustrating the pooled prevalence of mood stabiliser use*


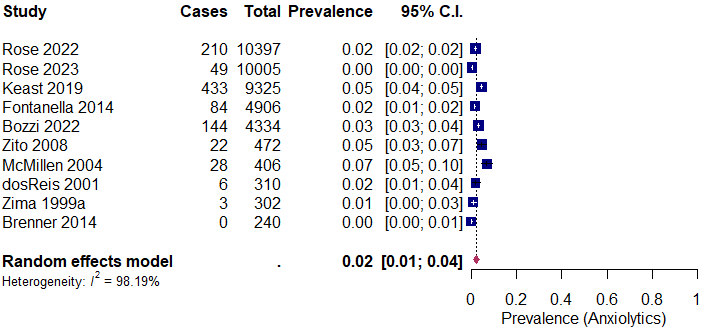


*Figure S5. Forest plot illustrating the pooled prevalence of anxiolytic use*
